# Native soil microorganisms hinder the soil enrichment with antibiotic resistance genes following manure applications

**DOI:** 10.1038/s41598-019-42734-5

**Published:** 2019-05-01

**Authors:** Eduardo Pérez-Valera, Martina Kyselková, Engy Ahmed, Frantisek Xaver Jiri Sladecek, Marta Goberna, Dana Elhottová

**Affiliations:** 1 Biology Centre of the Czech Academy of Sciences, Institute of Soil Biology, Na Sádkách 7, 370 05 České Budějovice, Czech Republic; 2 Biology Centre of the Czech Academy of Sciences, Institute of Entomology, Branišovská 31, 370 05 České Budějovice, Czech Republic; 3 Faculty of Science, University of South Bohemia, Branišovská 31, 370 05 České Budějovice, Czech Republic; 4 Department of Environment, Instituto Nacional de Investigación y Tecnología Agraria y Alimentaria (INIA). Carretera de la Coruña, Km 7.5, E-28040 Madrid, Spain

**Keywords:** Soil microbiology, Microbial communities, Microbial ecology

## Abstract

Bacterial genes responsible for resistance to antibiotic agents (ARG) are spread from livestock to soil through application of manure, threatening environmental and human health. We investigated the mechanisms of ARG dissemination and persistence to disentangle i) the influence of nutrients and microorganisms on the soil tetracycline (TET) resistome, and ii) the role of indigenous soil microbiota in preventing ARG spread. We analysed short-term (7 days) and persistent (84 days) effects of manure on the resistome of three antibiotic-free pasture soils. Four microcosm treatments were evaluated: control, mineral nutrient fertilization, and deposition of a layer of fresh manure onto soil or γ-irradiated soil. We quantified five TET-resistance genes, isolated 135 TET-resistant bacteria and sequenced both culturable TET-resistant and whole bacterial communities. Manure amendments, but not nutrient addition, increased the abundance of TET-r genes such as *tet*(Y). Such changes persisted with time, in contrast with the TET-resistant bacterial composition, which partially recovered after manure amendments. Manured γ-irradiated soils showed significantly lower nutrient content and higher TET-r gene abundance than non-irradiated soils, suggesting that native soil bacteria are essential for the fertilization effect of manure on soil as well as control the dissemination of potentially risky TET-r genes.

## Introduction

The increasing prevalence of antibiotic resistance among bacteria is a global clinical problem^1^ that has an important ecological dimension. The massive and widespread use of antimicrobial agents in humans and animals (e.g. livestock) selects for resistant microorganisms^2^ that enter the environment and the food chain. Organic wastes such as municipal sewage sludge and animal manure, containing high levels of antibiotic-resistant bacteria (ARB) and antibiotic resistance genes (ARG), are commonly used for agricultural fertilization. Manure application to land represents one of the main routes for spreading ARB along with ARG to the environment^3^. Although the survival of ARB in manured soils can be limited, it has been shown that ARG are able to persist in manured soils in the long term^4,5^. There has been an increasing concern about the accumulation of ARGs in soils as they may be exchanged between soil bacteria and human pathogens^6^. ARG are hence nowadays recognized as an emerging environmental pollutant^7^. However, the factors contributing to the ARG dissemination and persistence in soil are not well understood yet.

Understanding the fate of ARG in manured soils is complicated by the complex biotic and abiotic interactions between manure and soil. Manure improves soil fertility by altering main physical and chemical properties of soil, such as structure, water content, pH or nutrient availability, and also the interaction among microbial cells by e.g. increasing water filtration^8^. Manure microorganisms are passively introduced into soil by water fluxes, although active movement of motile cells in water suspension might also occur over short distances^8^. Importantly, the survival rate of introduced bacteria determines their potential to contaminate soils with ARG^9^. The fate of manure-borne bacteria in the soil, however, depends on the abilities of the particular organism and the biological, physical and chemical conditions in soil existing prior to manure application^8^. Moreover, the soil conditions imposed by added manure may also determine the fate of manure bacteria^8^, as higher nutrient availability would prolong their survival^10^. Native soil bacteria seem to play a relevant role in survival and persistence of manure bacteria, as they prevent the establishment of invaders in the soil^11–13^. Competitive interactions between manure and soil bacteria such as fighting for limiting resources may drive the assembly of soil bacterial communities^11,12^, expecting native bacteria to be better adapted to soil environments and therefore more successful in the interaction^3,14^. Indeed, a recent study has shown that the interaction between manure and indigenous soil bacteria might be key in determining the outcome of ARG in soils^12^. In addition, the indigenous soil ARB and ARG may also respond to changing nutrient conditions in soil^15^.

Most studies that experimentally analysed the persistence of ARG in manured soils used a mixture of manure and soil to simulate the situation in which arable soils are fertilized by manure^4,12,16–18^. In this study, we have focused on the situation in pasture soils, where cattle excrements (raw manure) remain deposited on the surface of soil, rather than being mixed with the upper soil layer. The natural deposition of raw manure in both extensive (i.e. pasture) and intensive (i.e. outdoor feedlot) production systems includes a scenario in which both biotic and abiotic components of manure move vertically, down into the ground. Despite experimental studies have previously explored how manure nutrients move into the soil^19^, soil enrichment with ARB and ARG and their persistence in the manure-soil interface under this approach remain poorly understood.

Our work focused on tetracycline antibiotics (TET), which belong to the most commonly used classes of antibiotics in agriculture, aquaculture and the clinic, being important pollutants of agricultural soils^3^. Microbial resistance to TET is an ancient natural phenomenon that existed before human use of antibiotics^20^ and involves diverse mechanisms such as efflux pumps, ribosomal protection proteins or enzymes for TET inactivation^21,22^. In previous studies we showed that the gut microbiome of dairy cows was a significant reservoir of TET-resistance (TET-r) genes regardless of whether the animals were under prophylactic or therapeutic treatment, or had never been treated with TET^4,16^. In addition, we found that dairy cow manure contained diverse TET-r genes that were transferable to soil under control laboratory conditions^4,16^ and which also persisted in soil under field conditions^17^. As the survival of TET-r gene hosts in the environment can be limited, it has been suggested that horizontal gene transfer into native soil bacteria could be involved in the long-term persistence of TET-r genes in soil^2,16,23,24^. Both broad-host-range (e.g. IncP1 and IncQ) and narrow-host-range (e.g. LowGC-type) plasmids could be vectors introducing TET-r genes into native soil bacteria^25,26^. In our previous study, we also showed that cattle manure contained LowGC plasmids carrying the *tet*(Y) gene and that the relative abundance of LowGC plasmids and *tet*(Y) correlated *in situ*^23^. In this study, we comparatively assessed the effects of manure, soil abiotic properties, and soil microbiome on the outcoming TET resistome and soil nutrient status in pasture soils receiving fresh cattle manure.

**Figure 1:**
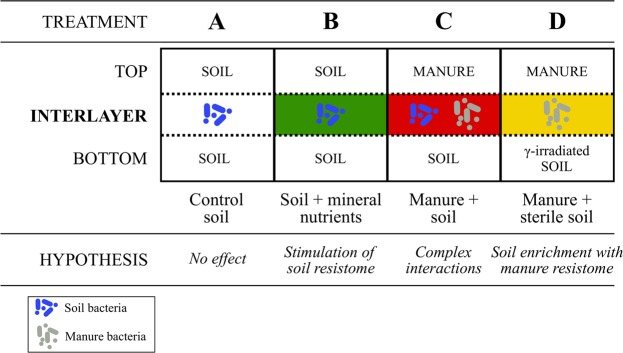
Schematic representation of the microcosm experiment. Each microcosm contained 3 layers (upper layer: 1 cm, middle layer (interlayer): 0.7 cm and bottom layer: 3 cm), the interlayer being separated from the top and bottom layers in all treatments with a sterile plastic net (dashed line). The top layer was established with either native soil (treatments A,B) or fresh manure (treatments C,D). The interlayer (A,C) and the bottom layer (A,B and C) were established with either native soil or γ-irradiated soil (D). The interlayer (B) was established with native soil amended with N-P-K. The expected interactions in the studied interlayer from three soils (S, B and M) are indicated as hypotheses. See the main text for further details.

We used experimental microcosms that enabled us to study the processes at the manure-soil interface layer, i.e. the specific soil region where most interactions responsible for soil enrichment with ARG from manure can be expected. Specifically, we assessed the short-term (7 days) and long-term (84 days) response of soil nutrient status, TET-r genes and LowGC plasmids (qPCR), and the TET-resistant bacterial subcommunities and total bacterial communities (Illumina sequencing of 16S rRNA genes) on soil amendments in four treatments (Fig. 1). The control treatment (A) contained native soil, treatment B native soil + mineral nutrients, treatment C native soil + fresh manure, and treatment D contained γ-irradiated soil + fresh manure. We used soils from three antibiotic-free farms (S, B and M soils, Table S1) and fresh manure from chlortetracycline-treated dairy cows (used in our previous studies^17,23^, see above). All microcosms were set up following a 3-horizontal-layer design, including either soil (treatments A and B) or manure (treatments C and D) in the top layer, and soil in the intermediate and bottom levels. Our study focused mainly on the intermediate layer, i.e. the contact interface between manure and soil.

## Results

### Effects of manure and mineral fertilizers on soil properties

Fresh dairy manure significantly altered main soil abiotic properties at the manure-soil interface, but these effects depended on treatment, time of incubation or on both (Fig. S1). Specifically, in contrast to the control treatment, manure increased soil pH and water content (treatments C and D) to levels close to fresh manure (mean ± SE, pH = 7.3 ± 0.1, water content = 86 ± 0.2%), and both remained elevated after 84 days. Interestingly, despite fresh manure contained high levels of carbon and other elements (C = 437 ± 0.7, N = 23.5 ± 0.2, P = 7.9 ± 0.2 mg g^−1^ dw), it only improved the content of N and P in the native soils (treatment C, Fig. S1). When comparing the manure-amended treatments (C and D), higher pH and lower P and N content was found in the γ-irradiated soils (D) compared to the native soils (C), even after 84 days. Finally, mineral nutrients (treatment B), which consisted of N-P-K fertilizer applied directly to the microcosm soil interlayer, significantly increased soil N and P levels, decreased the C/N ratio and showed no effect on pH, water content or C (Fig. S1). Overall, P levels tended to increase in soil with time.

### Effects of manure and mineral fertilizer on TET-r genes

**Figure 2:**
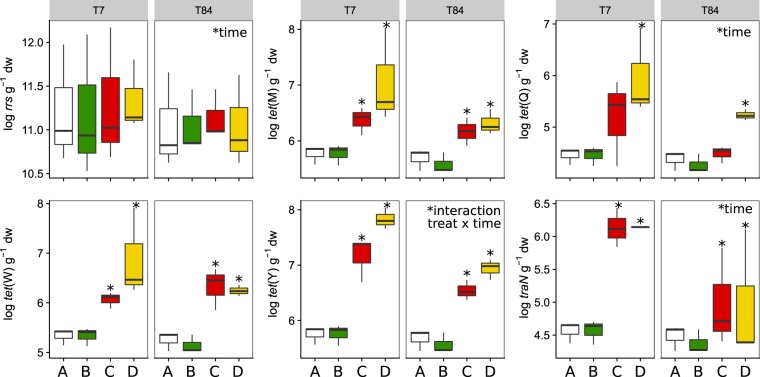
Boxplot of TET-r gene content (log copies per g^−1^ dry soil) in total DNA from the interlayer at 7 (T7) and 84 days (T84). Medians, upper and lower quartiles (boxes) and standard deviations (whiskers) were obtained from three soils, each measured in four technical replicates. Treatments are indicated as follows: control soil (A, white), soil + nutrients (B, green), manure + soil (C, red) and manure + γ-irradiated soil (D, yellow). Asterisks indicate significant differences (p < 0.05) between individual treatments and the control soil. Background levels of TET-r genes in A and B treatments were always below the limit of detection (LOD) and therefore, replaced by the corresponding LOD values. Differences between treatments C and D were significant for *tet*(Q) and *tet*(Y) in both time points.

Fresh manure harboured high abundance of bacteria (mean ± SE, 16S rRNA gene = 13.2 ± 0.02 log copies g^−1^ dw), TET-r genes (*tet*(M) = 7.9 ± 0.01; *tet*(W) = 9.4 ± 0.01; *tet*(Y) = 6.9 ± 0.00; *tet*(Q) = 8.7 ± 0.01) and LowGC plasmids (*traN* = 5.8 ± 0.04), which were eventually enriched in the manure-soil interface as shown in Fig. 2. In particular, compared to the control in which TET-r genes and LowGC plasmids remained below the limit of detection (see Materials and Methods), manure increased the abundance of *tet*(M), *tet*(W), *tet*(Y) and *traN* genes at the manure-soil interface (treatments C and D) over the three months of incubation. The effects of manure on the soil resistome depended on the presence of native soil bacteria, as *tet*(M), *tet*(W), *tet*(Y) and *traN* increased in both manure-amended treatments, while *tet*(Q) significantly increased only in the γ-irradiated soils. No stimulatory effect of mineral nutrients on TET-r genes (treatment B) was found.

The efficiency of gene enrichment in soil was between 60–114% at T7 and between 52–101% at T84 (Table 1). The values over 100% were recorded for the *tet*(Y) and the *traN* genes at 7 days, implying that the abundances of *tet*(Y) and *traN* at the manure-soil interface transiently exceeded those in fresh manure.

**Table 1:** Efficiency (in percentage) of gene enrichment in soil following manure application per treatment and time point, calculated as the ratio between the abundance of TET-r genes in treatments C and D, and fresh manure.

Time	Treatment	Genes				
		tet(M)	tet(W)	tet(Q)	tet(Y)	traN
7 days	C	81	65	60	104	106
	D	90	73	69	114	106
84 days	C	79	67	52	95	86
	D	80	66	60	101	86

The content of *tet*(Y) remained at the levels of fresh manure even after 3 months. The bacterial load, *tet*(Q) and *traN* also decreased with time, however, most of the TET-r genes in manure-amended soils remained above the control soil levels after 3 months.

### Bacterial hosts of TET-r genes

**Figure 3:**
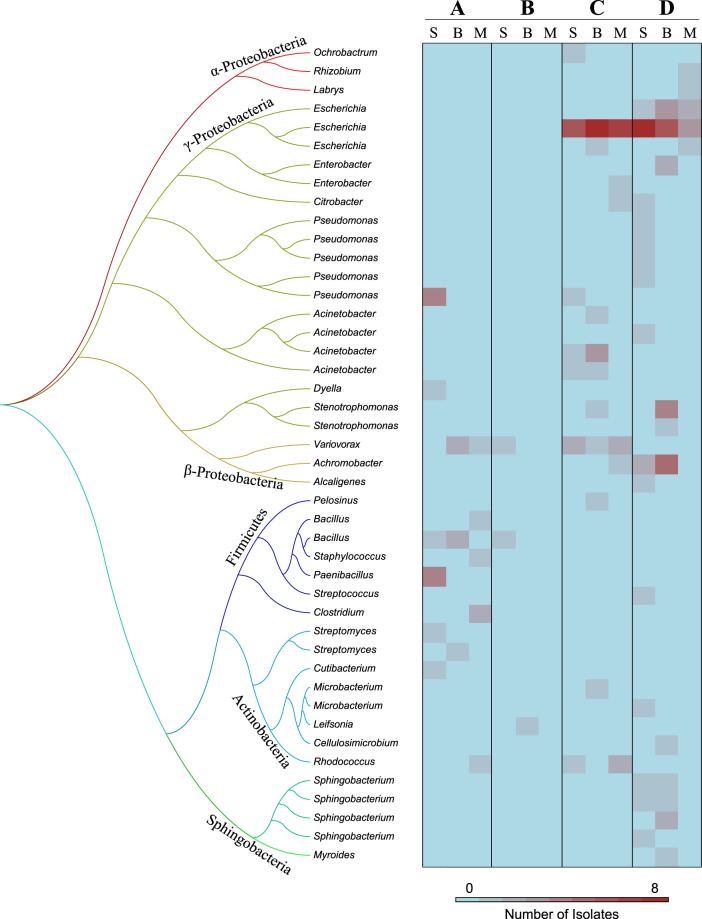
Maximum-likelihood molecular phylogenetic tree of TET-resistant bacterial strains isolated from three soils (S, B and M) under different treatments at 7 days. Treatments are indicated as follows: control soil (A), soil + nutrients (B), soil + manure (C) and γ-irradiated soil + manure (D). Colour intensity indicates the number of isolates.

A total of 135 bacterial strains able to grow in presence of 30 mg L^−1^ TET were isolated and identified from fresh manure and soil at the manure-soil interface after 7 days (Fig. 3; Table S2). Six TET-resistant strains belonging to the genera *Acinetobacter* (n = 3), *Escherichia* (n = 2) and *Cutibacterium* (n = 1) were isolated from manure (Table S2). Interestingly, both *Acinetobacter* and *Escherichia*, which were not found in the control and mineral treatments, were isolated from soil at the manure-soil interface after 7 days (Fig. 3). While *Escherichia* was isolated from both manure-amended soils (treatments C and D), *Acinetobacter* was found mainly in the native soil (treatment C) (Fig. 3). Conversely, *Sphingobacterium*, which was not found in fresh manure, was isolated only from the γ-irradiated soil (treatment D, Fig. 3). Finally, several bacterial genera found in the control soil, including *Bacillus* and *Paenibacillus* (Firmicutes), *Dyella* and *Pseudomonas* (Proteobacteria) and *Cutibacterium* and *Streptomyces* (Actinobacteria), were not captured at the manure-soil interface 7 days after manure amendment.

TET-resistant isolates were screened for the presence of *tet*(A), *tet*(M), *tet*(O), *tet*(Q), *tet*(W), *tet*(X) or *tet*(Y) genes (Table S2). The presence of *tet*(Y) was confirmed for several strains of *Acinetobacter*, *Escherichia*, *Pelosinus* and *Rhizobium*, *tet*(O) for *Variovorax*, *Sphingobacterium* and *Stenotrophomonas* and *tet*(X) for *Sphingobacterium* (Table S2).

### TET-resistant bacterial subcommunities

TET-resistant bacterial subcommunities were obtained from the S soil after cultivation of bacterial fractions in medium with 30 mg L^−1^ TET (three technical repetitions, see Material and Methods). The sequencing of TET-resistant subcommunities (16S rRNA genes), used to describe the bacterial taxonomic composition, revealed 728 OTUs, of which 184 were found in fresh manure (Fig. S2). Compared to the control, manure-amended soils had more TET-resistant OTUs after 7 and 84 days, although the differences decreased over time (Fig. S2).

Sequence analysis showed that fresh manure harboured a unique TET-resistant subcommunity dominated by three OTUs belonging to the genera *Providencia* (relative abundance 40 ± 3%), *Enterococcus* (40 ± 3%) and *Escherichia-Shigella* (13 ± 1%) that were not detected in the control soil. OTUs from manure enriched the soil differently depending on the treatment (Fig. S3). Unlike the control that was mainly composed of *Dyella*, *Providencia* was the dominant genus in all manure-amended soils followed by *Enterococcus* and *Alcaligenes* in the native soil (treatment C) and *Achromobacter* and *Vagococcus* in the γ-irradiated soil (treatment D) after 7 days (Fig. S3). After 3 months, *Variovorax* became the dominant genus in both manure-amended soils, although *Dyella* partially recovered its abundance in the native soil.

### Total bacterial communities

The number of OTUs of total bacterial communities, obtained from soil DNA isolated from the S soil (three technical repetitions), was 10,944, of which 1,444 OTUs were found in fresh manure (Fig. S4). Both manure-amended soils (C, D) showed lower number of OTUs compared to the control at T7, with opposite trends at T84, as native and γ-irradiated soils had respectively higher and lower number of OTUs with respect to the previous time point (Fig. S4).

Bacteria from fresh manure mainly belonged to Bacteroidetes (45 ± 0.5%) and Firmicutes (37 ± 0.3%). After 7 days, Proteobacteria and Actinobacteria dominated in the interlayer of all microcosms, Proteobacteria being the predominant taxa in the manure-amended γ-irradiated soil (Fig. S5). Interestingly, while the dominant families or genera at the native manure-soil interface remained mainly unknown, those found in the γ-irradiated soil belonged mainly to Burkholderiaceae such as *Comamonas* (15 ± 1%), Xanthomonadaceae such as *Stenotrophomonas* (11 ± 1%), or Sphingobacteriaceae such as *Sphingobacterium* (9 ± 1%) after 7 days. *Acinetobacter* (3 ± 0.1%) was less abundant but also relevant in the γ-irradiated soil after 7 days. Proteobacteria dominated in both manure-amended soils after 84 days while Actinobacteria became the most abundant phylum in the control and mineral-nutrient amended soils (Fig. S5).

### Ordination analyses

**Figure 4:**
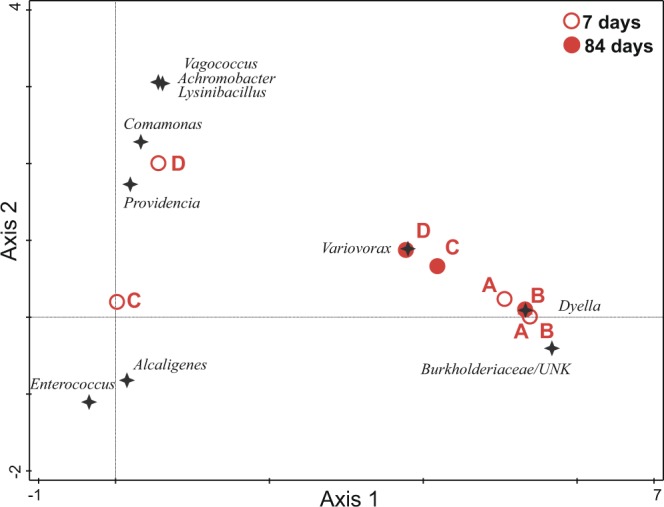
DCCA ordination diagram of TET-resistant subcommunities from the S soil. The time × treatment combinations are represented by red circles (open circles for T7 and filled circles for T84). Bacterial OTUs with the highest relative weight in the analysis (n = 10, see the main text for further details) are shown as black stars. OTUs are labelled according to their genus classification (UNK = unknown organism).

**Figure 5:**
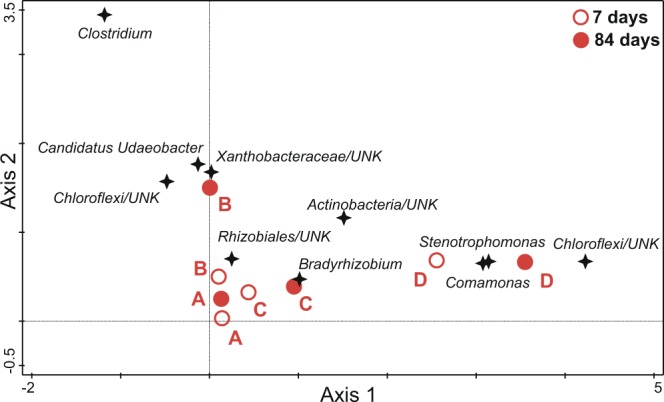
DCCA ordination diagram of total bacterial communities from the S soil. The time × treatment combinations are represented by red circles (open circles for T7 and filled circles for T84). Bacterial OTUs with the highest relative weight in the analysis (n = 10, see the main text for further details) are shown as black stars. OTUs are labelled according to their genus classification (UNK = unknown organism).

Both TET-resistant bacterial subcommunities (F = 64.4, p < 10^−3^, all canonical axes explain 96.57% of variability in data) and total bacterial communities (F = 9.92, p < 10^−3^, all canonical axes explain 80.07% of variability in data) were significantly structured by combination of treatment and time in the studied soil. The composition of TET-resistant and total bacterial communities was clearly affected by the addition of manure. That is, distinct microbial assemblages were formed in comparison to soils without manure (treatments A and B), which showed similar composition in both TET-resistant and total communities, and throughout both incubation times (Figs 4 and 5). The two types of studied communities from the manure-soil interface displayed, however, opposite development with time. Concerning the TET-resistant subcommunities in manure-amended treatments (C and D), the distance from A and B treatments decreased over time. In contrast, the total bacterial communities in C and D became more distant from A and B with increasing time. In the case of treatment C, however, this trend was much less pronounced.

## Discussion

Our results showed that cattle manure, but not mineral nutrients, altered the soil tetracycline resistome in laboratory microcosms by increasing the abundance of TET-r genes. By using a novel experimental design in which manure was spatially delimitated in a way that mimics the natural manure deposition, the effects of both abiotic and biotic components of manure on the soil resistome and bacteriome were studied, including the potential dissemination of antibiotic resistance genes in γ-irradiated soils. Our model allowed us to observe the impact of manure on soil properties, soil resistome and bacteriome in the specific contact interface layer. Our results suggest that native soil bacteria may hinder the spreading of antibiotic resistance genes while enhancing soil fertility after manure application.

Manure affected soil properties in adjacent soil layers as soon as 7 days after application. The elevated water content of manure (ca. 86%) rapidly reached the manure-soil interface layer, allowing the soil enrichment with nutrients, bacteria and genes. Interestingly, the fertilization effect of manure on soil, including improvement of the soil N and P content, was only found in the native soil and not in the γ-irradiated soil. Gamma radiation may alter soil structure and properties such as the stability of the soil aggregates, altering the water percolation^27,28^. Our results showed, however, that γ-radiation did not alter the main soil physical and chemical properties (as analysed before setting up the microcosms), nor water percolation, as water content was similar to native soils at both times. It seems, therefore, that native soil microorganisms might influence the availability and movement of nutrients from manure to soil. Autochthonous soil microorganisms are functionally diverse and have key roles in nutrient cycling and soil fertility^29^, which could be particularly relevant after manure amendments^30^.

Manure improved soil abiotic properties but increased the risk of dissemination of TET-r genes, plasmids and bacteria into the soil. Specifically, over the 84-day incubation period, we found high levels of *tet*(M), *tet*(W), *tet*(Y) and LowGC plasmids at the manure-soil interface in all manure-amended treatments, and additionally *tet*(Q) in the γ-irradiated soils. The genes most likely originated from manure, as their levels in control soils remained below detection limits and did not respond to the addition of mineral nutrients. While the TET-resistant subcommunities in the control soil were represented by a few taxa dominated by *Dyella*, the manure-soil interface harboured a rich TET-resistant bacteriome dominated by *Providencia* and *Enteroccoccus* in the case of native soils, and by *Providencia* and *Achromobacter* in the case of γ-irradiated soils, already 7 days after addition. These genera have been frequently found in cow and pig manure and may harbour TET-r genes such as *tet*(W), *tet*(Q) or *tet*(M)^31–34^. While high levels of TET-r genes persisted in soil over the whole incubation period, the composition of TET-resistant subcommunities partially reverted at the end of the experiment. In particular, the TET-resistant subcommunity at the manure-soil interface was replaced by less-rich subcommunities dominated by *Variovorax* and *Dyella* at 84 days, which were not found in fresh manure. This suggests that indigenous bacteria may capture and keep TET-r genes from manure, which is consistent with previous findings suggesting that ARG persist in the environment regardless of the survival of the primary host^3^.

The impact of manure on total bacterial communities at the manure-soil interface was negligible in the case of native soils, compared to that in γ-irradiated soils. In addition, the identified TET-resistant subcommunity represented only 18% of total bacterial community of the native soil at T7, estimated as the relative abundance of OTUs shared between the two types of communities, while it accounted for as much as 69% of the total community in the case of γ-irradiated soil. This data suggests that the indigenous soil bacteria in the upper soil layer may act as a barrier against TET-resistant species from manure. This applies also on TET-r genes as the content of *tet*(Y) and *tet*(Q) at the manure-soil interface of γ-irradiated soil was higher, compared to native soils. Soil microorganisms, therefore, may hinder the entrance of certain TET-r genes from manure to the upper soil layer, most likely by preventing the establishment of their bacterial hosts. Comparable results were found for mixtures of manure or biogas digestates with soil in microcosm experiments suggesting that increased competition for nutrients, antibiosis or smaller niche availability prevent the establishment of TET-resistant or pathogenic bacteria from the applied organic fertilizers^12,13^. All these studies also used γ-radiation to evaluate the role of indigenous soil bacteria in controlling the establishment of exogenous bacteria in soil. γ-radiation is considered as a reliable method that produces low soil disruption compared to other methodologies, being the most used and prospective technique for selective elimination of target organisms in soil ecological studies^12,13,27,28,35,36^. Indeed, we did not detect any artefact caused by γ-radiation in our study. The possibility of increased transfer of bacteria and ARG from manure into soil due to soil conditions imposed by γ-radiation, however, cannot be totally excluded. More detailed and extensive analyses including stability of soil microaggregates, which could alter the water percolation^27^ and potentially enhance the transfer of bacteria and ARG from manure into soil, merit further attention. In addition, analyses of a broader spectrum of nutrients and micronutrients would be also helpful, since γ-radiation may increase the NH_4_^+^-N concentration in soil, which may facilitate the growth of exogenous bacteria^13^.

Despite the abundance of TET-r genes increased in manured soils compared to control, their levels remained, in general, lower than those in fresh manure. However, an opposite trend was observed for *tet*(Y), with higher abundances in manured soil compared to fresh manure. Assuming that every gene has its own range of hosts^23,37–39^, the results indicate that bacteria harbouring *tet*(Y) could have been favoured at the manure-soil interface. Although the bacterial hosts of *tet*(Y) in fresh manure could not be identified, several strains of *Acinetobacter*, *Escherichia*, *Pelosinus* and *Rhizobium* were confirmed as hosts of *tet*(Y) at the manure-soil interface at 7 days. According to our sequencing data, at this time *Acinetobacter* was rare ( < 0.1%) within the TET-resistant subcommunity but became relevant (ca. 3.4%) in the total community in γ-irradiated soils. *tet*(Y) showed a similar trend, suggesting that *Acinetobacter* could disseminate TET-r genes in soil. In our previous study on the dairy farm, source of manure used in this study, *tet*(Y) showed a higher abundance in manure deposited on the farm floor, as compared to rectal samples^17^. This suggests that manure hosts of *tet*(Y), either *Acinetobacter* or others, may proliferate in aerobic conditions^17,18,40^. Alternatively, the presence of *tet*(Y) in non-faecal bacteria such as *Rhizobium* suggests a spread into indigenous soil bacteria via HGT. Interestingly, the abundance of LowGC plasmids, previously shown to harbour *tet*(Y)^23^, was also higher at the manure-soil interface, making LowGC plasmids candidates for HGT of *tet*(Y).

Members of *Dyella*, *Variovorax*, *Enterococcus* or *Sphingobacterium*, relevant before and/or after the manure amendment, could also implicate important risks for ARG dissemination. Environmental species of *Dyella* or *Variovorax* can deal with adverse conditions, being potential reservoirs of TET-r genes but also opportunistic pathogens^41–44^. For example, Kobashi *et al*.^43^ found *Dyella* to carry *tet*(Y) in forest soils. Santamaría *et al*.^32^ also detected *tet*(Q) and *tet*(W) in *Dyella* in grasslands with limited administration of antibiotics. In addition, they also found other TET-resistant non-clinical bacteria carrying *tet*(W) and *tet*(Q) genes such as *Variovorax*^32^, which in our study dominated in all manured soil in the long term. Notably, some TET-resistant bacteria from manure also became relevant in soil. For example, *Enterococcus* spp., dominant in native soils after 7 days and commonly found in cow and pig manure^31,32^, can potentially resist numerous antibiotics^45^. Finally, *Sphingobacteria*, which could degrade organic compounds^46^ including TET-r antibiotics encoded by *tet*(X)^47^, colonized the soil lacking indigenous bacteria. These taxa could replace the missing community of degraders in soil, but at the same time may also introduce ARG.

In conclusion, our results show that manure, but not the mineral nutrients in a similar concentration, increased the soil levels of TET-r genes over 3 months. Higher levels of TET-r genes and lower fertility in soils with reduced biological viability indicate that indigenous soil microorganisms are important for both improving the fertility and limiting the spreading of TET-r genes in soils amended with manure.

## Materials and Methods

### Fresh manure and soil sampling

Soil was sampled in May 2017 at three small cattle farms (S, B and M) located in the South Bohemian region of the Czech Republic (approx. 48°North, 14°East). In these farms, the livestock load per unit of soil area was between 0.4–1.0 livestock unit, i.e. within the range of the Order of the Government of the Czech Republic (75/2015 Sb., 76/2015 Sb.). Soil in the farms is exposed to cattle for a maximum of 6 months during the growing season. Antibiotics are administered rarely, only as therapy in the case of serious health complications. Animals on all farms were treated by antiparasitic agents, either by *Aldifal* (active compound albendazole) on the B and S farms or by *Biomectin* (active compound ivermectin) on the M farm.

Soil samples were taken at 5–15 cm depth in duplicates in ten 10 × 10 m plots along a linear 200 m transect. Soil was sieved (<5 mm), pooled per farm and transported in an icebox to the laboratory. Soil was stored at 4 °C until the set-up of the microcosms. One week before starting the experiment, soil samples were pre-incubated at 25 °C in the dark. For soil characteristics, see Table S1.

Cattle excrement samples (“fresh manure”) were collected at a private dairy farm located in South Bohemia, where we previously studied the impact of prophylactic administration of chlortetracycline to cattle on dissemination of TET-r genes^17^. Chlortetracycline is used at this farm either prophylactically to prevent bacterial infections after calving but also to treat local traumas and other inflammatory processes of the extremities. Further details on the farm can be found in Kyselková *et al*.^17^. Samples of fresh manure were taken from 20 adult animals (3–7 years old) as an anal grab using a sterile glove to prevent contamination. Samples were collected the day of setting up the microcosms and taken to the laboratory for immediate use. After homogenization in one sample, aliquots were immediately separated for subsequent physical and chemical, bacteriological and genetic analyses.

### Microcosm assembly

Soil microcosms were established in plastic containers (volume 300 mL, base diameter 8 cm, upper diameter 9.5 cm, height 6 cm) in a three-horizontal layer design (Fig. 1). Our study focused on the interlayer (“manure-soil interface”), which was spatially delimited from the upper and bottom layers through a durable, autoclaved plastic net (mesh size 1.4 mm, glass fibre material of diameter 0.28 mm). Microcosms were settled by sequentially adding soil to the bottom layer (ca. 3 cm thick, 120 g), soil to the interlayer (ca. 0.7 cm thick; 60 g) and either soil or manure to the top layer (ca. 1 cm thick; 100 g), depending on the treatment (Fig. 1). The control treatment (A) contained unaltered soil in all three layers while we modified the substrate or the conditions in the other treatments as follows. A mineral-nutrient treatment (B) was established by adding nitrogen-phosphorous-potassium (N-P-K, in concentrations like those found in dry manure, i.e. 1.2% N, 0.2% P, 0.9% K) to the interlayer. Treatment C contained fresh manure in the top layer, and unaltered soil in the middle and bottom layer. Treatment D contained fresh manure in the top layer and γ-irradiated soil in the middle and bottom layer. The γ-irradiation procedure did not alter the main soil properties (Supplementary Methods).

Every microcosm combination (3 farm soils × 4 treatments) were replicated twelve times, making a total of 144 plastic containers that were incubated at 25 °C in the dark. The microcosms were incubated in randomized blocks and covered with a perforated lid that allowed aeration. Six replicates per microcosm combination were destructively sampled after 7 days incubation, the remaining after 84 days. Water was not added to the microcosms in order to simulate natural field conditions and not to alter the manure effect on soil moisture. The interlayer from all treatments was carefully separated from the other layers during sampling, combined across replicates and divided into aliquots (ca. 2 g) that were stored at −80 °C for DNA extraction. Isolation of TET-resistant bacterial strains was performed immediately after sampling. Manure and soil physical and chemical properties were analysed in triplicate following standard procedures (Supplementary Methods). Average values from technical replications were calculated for further statistics.

### DNA extraction and qPCR-based characterization of TET resistome

Total DNA was extracted in duplicate from ca. 0.5 g fresh manure or soil from the interlayer with the FastDNA SPIN kit for Soil (MP Biomedicals Europe, Illkirch, France) (Supplementary Methods).

The quantitative PCRs were done for the genes *rrs* (bacterial 16S rRNA), *tet*(Y) (coding for TET efflux pumps), *tet*(M), *tet*(Q) and *tet*(W) (ribosomal protection against TET), and *traN* (LowGC plasmid transfer). The quantified TET-r genes were previously shown to occur occasionally or regularly in the studied cattle manure^17^. The qPCRs were performed in quadruplicates using SYBR-Green detection and TaqMan probes, as described previously^16,17^ (Supplementary Methods). Prior to the quantification, the optimal dilution of template DNA was assessed as described in Kyselková *et al*.^17^, in order to avoid the inhibition of qPCR reactions. Limits of detection (LOD) and quantification (LOQ) were assessed as in Kyselková *et al*.^17^ (Supplementary Methods). Gene abundances below LOD or above LOQ were respectively replaced by the corresponding LOD and LOQ values. Gene abundances were log-transformed and averaged across technical replicates for further statistics.

### Isolation of TET-resistant bacteria and PCR screening

Bacteria were isolated from fresh manure at T0 and from soil at the manure-soil interface at T7 by the serial plate dilution method, using three different media and both aerobic and anaerobic conditions (Supplementary Methods). A total of 1,377 bacterial colonies were isolated and tested for growth on fresh plates supplemented with tetracycline (30 mg L^−1^), of which 135 TET-resistant cultures were selected for subsequent characterization (Table S2). The presence of TET-r genes in the isolates, including *tet*(A), *tet*(Y), *tet*(M), *tet*(O), *tet*(Q), *tet*(W) and *tet*(X), was checked via PCR and confirmed with the PCR product sequencing. The TET-resistant isolates were putatively identified with sequencing of their 16S rRNA genes, which were also used to reconstruct their phylogenetic relationships (Supplementary Methods). DNA sequences from 16S rRNA genes were deposited in GeneBank (Accessions MH725638 to MH725785).

### High-throughput sequencing of TET-resistant subcommunities and total bacterial communities

Only the microcosms established with soil from the S farm were chosen for the high-throughput sequencing of TET-resistant subcommunities and total bacterial communities at 7 and 84 days. Three technical replications were performed over the pooled soil per treatment and time. TET-resistant subcommunities were obtained by centrifugation of sample suspensions in Nycodenz gradient and subsequent enrichment of TET-resistant bacteria in tryptic soy broth supplemented with TET (30 mg L^−1^) (Supplementary Methods). Genomic DNA from TET-resistant cultures was extracted using DNeasy Blood & Tissue Kit (Qiagen, Germany) following the manufacturer instructions. Quality check and quantification of DNA was performed as for soil DNA.

High-throughput sequencing (Illumina platform) of 16S rRNA gene fragments amplified from DNA extracted from TET-resistant cultures and total soil DNA produced 1,208,633 sequences. Details on sequencing and sequence processing are described in Supplementary Methods. After the initial processing, 936,509 sequences were grouped into 10,581 OTUs. OTU abundances were standardized by dividing the number of reads per OTU between the total number of reads per sample. The relative abundance of each OTU was corrected by the estimated 16S rRNA gene copies, according to Kembel *et al*.^48^. Data from DNA sequencing was used to describe the taxonomic composition of bacterial groups present. The raw sequence data have been deposited in the GenBank SRA database (https://www.ncbi.nlm.nih.gov/bioproject/PRJNA482507).

### Statistical analyses

Treatment and time effects on soil abiotic properties and TET-r gene abundances were tested with generalized linear mixed models (GLMM) using the nlme package^49^ for R^50^. First, to explore the effect of manure on soil at the soil-manure interface, we performed a series of GLMMs with soil abiotic properties (e.g. pH) or TET-r genes as response variables and treatment and time as independent factors. Then, we tested the interaction effect between both factors. All models included soil origin (i.e. farm) as a random variable. Significant differences between treatments with respect to the control, and between C and D treatments, were tested through post-hoc pairwise comparisons using the function glht in the multcomp^51^ R package. Venn diagrams accounting for shared OTUs between treatments were constructed with the VennDiagram package^52^ for R.

The changes in bacterial compositions among treatments were analysed using detrended canonical analysis (DCCA), with detrending by second-order polynomial, in Canoco 5^53^. DCCA enabled us to analyse the response of a virtually unlimited number of species to a predictor in a single analysis, thus avoiding fitting a model for each species separately and eliminating the ‘arch effect’ artefact. Two separate DCCAs were run for bacterial communities, one for TET-resistant subcommunities and one for total communities. In both analyses, the OTU relative abundances were the response variables and treatment in interaction with sampling time the predictors. Statistical significance was assessed using Monte Carlo permutation tests (999 permutations). Finally, the OTUs with the highest weights in respective DCCAs were displayed in the final ordination diagrams. Interpretations were aimed primarily at the distances between treatment type × time combinations.

## Supplementary information

Supplementary Methods
