# Supplementary material for: Native soil microorganisms hinder the soil enrichment with antibiotic resistance genes following manure applications
Source: Sci Rep. 2019 May 1;9:6760. doi: 10.1038/s41598-019-42734-5 (PMC6494816; doi:10.1038/s41598-019-42734-5)
Supplement: Supplementary file 1 — Supplementary Methods [file 41598_2019_42734_MOESM1_ESM.pdf]

*Supplementary Information*

# Native soil microorganisms hinder the soil enrichment with antibiotic resistance genes following manure applications

Eduardo Pérez-Valera, Martina Kyselková, Engy Ahmed, Frantisek Xaver Jiri Sladecek, Marta Goberna & Dana Elhottová

## INDEX


- SUPPLEMENTARY METHODS……………………………………………………………2
- SUPPLEMENTARY TABLES……………………………………………………………8
- SUPPLEMENTARY FIGURES…………………………………………………………15

## SUPPLEMENTARY METHODS

### *Soil γ-irradiation*

Soil was treated by 76.8 kGy γ-radiation (1.6 kGy $h^{-1}$: two 24-hour-cycles with 3-days delay between individual cycles) from a $^{60}Co$ source (Research Centre Řež, Czech Republic). Soil sterility was checked by enumeration of total microscopic counts (TMC (Bloem, 1995)) and heterotrophic bacteria by the most probable number (MPN) plate count technique (Alexander, 1982). The chosen γ-radiation procedure was selected as the most effective (97% and 100% reduction of TMC and cultivable viable forms respectively) based on pilot experiments performed under different γ-radiation regimes. In addition, γ-radiation significantly reduced the 16S rRNA PCR templates in ca. 1 order of magnitude (paired t-test, $t=27.3$, $p=0.001$). The main soil properties were not altered by the γ-irradiation procedure (paired t-test; pH $t=0.4$, $p=0.7$; water content $t=-1.6$, $p=0.2$; carbon $t=0.54$, $p=0.65$; nitrogen $t=0.73$, $p=0.54$; phosphorous: $t=0$, $p=1$; carbon/nitrogen ratio $t=-2.02$, $p=0.2$, d.f.=2). Soil was used for the set up of the microcosms 48 hours after γ-irradiation.

### *Soil physical and chemical analyses*

Physical and chemical properties of manure and three soils used in this study were analyzed as in Kyselková et al. (2015b). Total C and N concentrations were determined by dry combustion on elemental analyser (vario MICRO cube, Elementar GmbH, Germany). Total P was measured colourimetrically by the ammonium molybdate-ascorbic acid method on a flow injection analyser (FIA, Lachat QC8500, Lachat Instruments, USA) after perchloric acid digestion (Kopáček and Hejzlar, 1995).

### *TET-resistant bacteria isolation, PCR screening and identification*

Bacteria were isolated from fresh manure and soil at the interlayer by the serial plate dilution method as described by Kyselková et al. (2015a). We prepared plates with Endo agar (Both DifcoTM; Becton, Dickinson and Co., USA) to isolate enteric bacteria, Tryptic soy agar for aerobic bacteria, CHROMagar™ for *Acinetobacter* and Schaedler agar (prepared plates purchased from DULAB s.r.o, Czech Republic) for anaerobic bacteria. All plates were inoculated

with 0.1 mL of manure or soil suspensions diluted to $10^{-1}$-$10^{-6}$. We cultivated CHROMagar and tryptic soy agar plates at 28 ºC for 24 hours and 7 days, and those of Endo agar and Schaedler at 37 ºC for 24 hours and 14 days, respectively. Schaedler plates were placed into anaerobic jars with Anaerocult A and Anaerotest (all Merck KGaA, Germany) to maintain anaerobic conditions. Representative colonies were picked up and purified in fresh plates. Fresh bacterial biomass was transferred to glycerol stocks (2 full bacteriological loops of biomass, 700 µL of tryptic soy broth + tetracycline (30 mg $L^{-1}$), 300 µL of 50 % glycerol) for long-term storage at -80 ºC.

The presence of TET-r genes in the isolates, including *tet*(A), *tet*(Y), *tet*(M), *tet*(O), *tet*(Q), *tet*(W), *traN* and *tet*(X) was checked via PCR after re-suspending one loop of bacterial cells in sterile water and three 5-min cycles of heating (95 ºC) and freezing (-20 ºC). PCR reactions (25 µL) contained 1 × KAPA Taq Ready Mix (KAPA Biosystems, Wilmington, MA), primers (Table S3) and DNA template (2 µL of bacterial lysates). Positive and negative (sterile water instead of DNA template) controls were included in every PCR run. Specificity of PCR products containing TET-r genes was confirmed by sequencing, as in Kyselková et al. (2015a).

PCR amplifications of 16S rRNA gene fragments were performed to identify the bacterial isolates. PCR reactions were carried out using the primers pA and pH according to Bruce et al. (1992). PCR-amplification products were purified using Illustra™ ExoProStar™ 1-step (GE Healthcare) and subjected to sequencing (Sanger dideoxy sequencing) at SEQme s.r.o. (Dobříš, Czech Republic). The obtained sequences were compared to sequences in the NCBI GenBank database using BlastN (Altschul et al., 1990). The recovered sequences were trimmed, grouped at 97% similarity and aligned with PYNAST (Caporaso et al., 2010a) in QIIME 1.9.1 (Caporaso et al., 2010b). Then, the hypervariable regions were removed. A phylogenetic tree was generated from the distance matrices using a maximum likelihood algorithm based on the Tamura-Nei model (Tamura and Nei, 1993). The phylogenetic tree was constructed in MEGA X (Kumar et al., 2018).

*Soil DNA extraction*

Total DNA was extracted in duplicate from fresh manure or the manure-soil interface with the FastDNA SPIN kit for Soil (MP Biomedicals Europe, Illkirch, France) according to the manufacturer instructions with one modification as follows. DNA bound to silica matrix was

washed in 1 ml guanidine thiocyanate (Sigma-Aldrich, Prague, Czech Republic; 5.5 M), as in Kyselková et al. (2015b). DNA quality was checked by electrophoresis in 1% agarose gels run in 1 × Tris–acetate–EDTA buffer. Extracted DNA was quantified by NanoDrop 2000 Spectrophotometer (Thermo Scientific, Wilmington, DE) and checked for the absence of PCR inhibitors by amplification of 16S rRNA genes (Table S3). DNA aliquots per soil, treatment and time were mixed for further analyses.

*qPCR assessment of TET-r genes and 16S rRNA genes*

TET-r genes and 16S rRNA were quantified as described previously (Kyselková et al., 2013; Kyselková et al., 2015a). Briefly, FastStart Universal SYBR Green Master (ROX; Roche, Basel, Switzerland) and 300 nM primers were used for *rrs*, *tet*(Q) and *tet*(W), and 800 nM primers for *tet*(Y). KAPA PROBE FAST ABI Prism qPCR Master Mix (KAPABiosystems) was used for quantification of *tet*(M) and *traN*, using 300 nM primers and TaqMan probes (See Table S3 for the primers and probes used). All qPCR reactions were performed on StepOne Plus Real-Time PCR system (Applied Biosystems, Foster City, CA). The specificity of qPCR was checked by inspecting PCR product melt curves (for SYBR Green assays) and by checking the length of the PCR products in agarose gels.

Limits of detection (LOD) and quantification (LOQ) were assessed for each TET-r gene as in Kyselková et al. (2015a). Briefly, we used two-fold dilutions of standards, which contained ca. $10^3$ to <1 copies in 6 replicates. We then estimated the LOD and LOQ from the obtained threshold cycle (ct) values with the sofware Genex Enterprise Version 6 (MultiD Analyses AB, Goteborg, Sweden). LOD (95% probability level for gene detection) were respectively 155.6, 59, 7.8, 160 and 10 gene copies per reaction for *tet*(Y), *tet*(W), *tet*(Q), *tet*(M) and t*raN* (LowGC). LOQ (number of gene copies resulting in less than 25% variation coefficient of ct) were respectively 674, 197, 56, 561 and 574 for *tet*(Y), *tet*(W), *tet*(Q), *tet*(M) and t*raN* (LowGC).

*Nycodenz gradient and TET culture enrichment*

Manure and soil microcosm samples were added to falcon tubes supplemented with 25 ml buffer (0.2M NaCl and 50 mL Tris-Cl) (Berry et al., 2003) and homogenized by shaking at 4 ºC. After soil and excrement particles were disrupted by vortexing, samples were centrifuged (700 rpm, 5

minutes at 4 ºC) (Marco, 2010) and supernatant from each tube was loaded on top of a 10 ml Nycodenz solution (80% w/v, PROGEN Biotechnik GmbH, Heidelberg, Denmark), which was prepared in ultrapure water, and sterilized by autoclaving. Prepared samples were centrifuged at 10 000 rpm (35 min, 4º C) and a volume of 5 ml of the supernatant (including the bacterial cells) was transferred to sterile falcon tubes with PBS buffer (phosphate buffered saline, pH 7.4) with a final volume of 35 ml. Samples were centrifuged at 10 000 rpm (10 min, 4 ºC) (Berry et al., 2003) and the pellets (i.e. cells) resuspended in PBS buffer. TET-resistant enriched subcommunities were acquired after cultivation of bacterial suspension ($1\times10^9$ of bacterial cells per ml media, estimated by fluorescence microscopy using 0.01% DAPI) in tryptic soy broth supplemented with tetracycline (30 mg $L^{-1}$) and cycloheximide (100 mg $L^{-1}$) (Huang et al., 2014). Samples were aliquoted and stored at -80 °C until the DNA extraction was performed.

*High-throughput sequencing of TET-resistant subcommunities and complete bacterial communities*

TET-resistant subcommunities and total bacterial communities were characterized by amplification and high-throughput sequencing of 16S rRNA V4 gene fragments using the Illumina platform and the primers 515F (5'-GTGCCAGCMGCCGCGGTAA-3') and 806R (5'-GGACTACVSGGGTATCTAAT-3') (Caporaso et al., 2012). Each sample contained a unique barcode and a two-base linker (GT or CC) before the primer. PCR amplifications were performed using 5 µL 5xQ5 buffer, 0.5 µL PCR Nucleotide Mix (10 mM), 1.5 µL BSA (10 mg $mL^{-1}$), 0.25 µL Q5 High-Fidelity DNA polymerase, 1 µL forward primer (10 pmol $\mu L^{-1}$), 1 µL reverse primer (10 pmol $\mu L^{-1}$), 5µ 5xQ5HighGC Enhancer, 1 µL DNA template (approximately 5-50 ng) and $H_2O$ to a total volume of 25 µL. PCR conditions were as follows: 4 min at 94 °C, 25 cycles of 30 s at 94 ºC, 1 min at 50 ºC and 75 s at 72 ºC, followed by 10 min at 72 ºC. PCR products were mixed in equal concentrations and purified using MinElute PCR Purification kit (Qiagen, Hilden, Germany). Sequencing was performed at the Institute of Microbiology (Prague, Czech Republic) using Illumina MiSeq with v3 chemistry.

*Sequence processing*

Sequence processing was conducted using SEED 2 (Větrovský et al., 2018) as follows. Initially, paired ends were joined with the fastq-join tool (Aronesty, 2013) and short (<200 bp), low quality

(Phred score < 30) or those sequences with ambiguous base calls (Ns) removed. Primers and barcodes were trimmed after sequence de-multiplexing. Chimeric sequences were removed and operational taxonomic units (OTUs) clustered at an identity level of 97% using USEARCH (Edgar, 2013). OTUs were taxonomically classified using BLAST and the SILVA database (Release 132 (Quast et al., 2013) in QIIME 1.9.1 (Caporaso et al., 2010)).

## References


Alexander, M., 1982. Most probable number method for microbial populations, in: Page, A.L., Miller, R.H., Keeney, D.R. (Eds.), Methods of Soil Analysis, Part II: Chemical and Microbiological Methods. American Society of Agronomy, Madison, WI, USA, pp. 815–820.

Altschul, S.F., Gish, W., Miller, W., Myers, E.W., Lipman, D.J., 1990. Basic local alignment search tool. Journal of Molecular Biology 215, 403–410.

Aminov, R.I., Chee-Sanford, J.C., Garrigues, N., Teferedegne, B., Krapac, I.J., White, B.A., Mackie, R.I., 2002. Development, validation, and application of PCR primers for detection of tetracycline efflux genes of gram-negative bacteria. Applied and Environmental Microbiology 68, 1786–1793.

Aronesty, E., 2013. Comparison of sequencing utility programs. The Open Bioinformatics Journal 7, 1–8.

Berry, A.E., Chiocchini, C., Selby, T., Sosio, M., Wellington, E.M.H., 2003. Isolation of high molecular weight DNA from soil for cloning into BAC vectors. FEMS Microbiology Letters 223, 15–20.

Bloem, J., 1995. Fluorescent staining of microbes for total direct counts, in: Akkermans, A.D.L., Van Elsas, J.D., De Bruijn, F.. (Eds.), Molecular Microbial Ecology Manual. Springer Netherlands, Dordrecht, pp. 1–12.

Bruce, K.D., Hiorns, W.D., Hobman, J.L., Osborn, A.M., 1992. Amplification of DNA from native populations of soil bacteria by using the polymerase chain reaction 58, 3413–3416.

Caporaso, J.G., Bittinger, K., Bushman, F.D., DeSantis, T.Z., Andersen, G.L., Knight, R., 2010a. PyNAST: a flexible tool for aligning sequences to a template alignment. Bioinformatics 26, 266–267.

Caporaso, J.G., Kuczynski, J., Stombaugh, J., Bittinger, K., Bushman, F.D., Costello, E.K., Fierer, N., Pena, A.G., Goodrich, J.K., Gordon, J.I., Huttley, G.A., Kelley, S.T., Knights, D., Koenig, J.E., Ley, R.E., Lozupone, C.A., McDonald, D., Muegge, B.D., Pirrung, M., Reeder, J., Sevinsky, J.R., Turnbaugh, P.J., Walters, W.A., Widmann, J., Yatsunenko, T., Zaneveld, J., Knight, R., 2010b. QIIME allows analysis of high-throughput community

sequencing data. Nature Methods 7, 335–336.

Caporaso, J.G., Lauber, C.L., Walters, W.A., Berg-Lyons, D., Huntley, J., Fierer, N., Owens, S.M., Betley, J., Fraser, L., Bauer, M., Gormley, N., Gilbert, J.A., Smith, G., Knight, R., 2012. Ultra-high-throughput microbial community analysis on the Illumina HiSeq and MiSeq platforms. The ISME Journal 6, 1621–1624.

Edgar, R.C., 2013. UPARSE: Highly accurate OTU sequences from microbial amplicon reads. Nature Methods 10, 996–998.

Huang, K., Tang, J., Zhang, X.X., Xu, K., Ren, H., 2014. A comprehensive insight into tetracycline resistant bacteria and antibiotic resistance genes in activated sludge using next-generation sequencing. International Journal of Molecular Sciences 15, 10083–10100.

Kopáček, J., Hejzlar, J., 1995. Semi-micro determination of total phosphorus in soils, sediments, and organic materials: A simplified perchloric acid digestion procedure. Communications in Soil Science and Plant Analysis 26, 1935–1946.

Kumar, S., Stecher, G., Li, M., Knyaz, C., Tamura, K., 2018. MEGA X: Molecular Evolutionary Genetics Analysis across Computing Platforms. Molecular Biology and Evolution 35, 1547–1549.

Kyselková, M., Jirout, J., Chroňáková, A., Vrchotová, N., Bradley, R., Schmitt, H., Elhottová, D., 2013. Cow excrements enhance the occurrence of tetracycline resistance genes in soil regardless of their oxytetracycline content. Chemosphere 93, 2413–2418.

Kyselková, M., Jirout, J., Vrchotová, N., Schmitt, H., Elhottová, D., 2015a. Spread of tetracycline resistance genes at a conventional dairy farm. Frontiers in Microbiology 6, 1–14.

Kyselková, M., Kotrbová, L., Bhumibhamon, G., Chroňáková, A., Jirout, J., Vrchotová, N., Schmitt, H., Elhottová, D., 2015b. Tetracycline resistance genes persist in soil amended with cattle feces independently from chlortetracycline selection pressure. Soil Biology and Biochemistry 81, 259–265.

Marco, D., 2010. Metagenomics: theory, methods, and applications. Caister Academic Press.

Quast, C., Pruesse, E., Yilmaz, P., Gerken, J., Schweer, T., Yarza, P., Peplies, J., Glockner, F.O., 2013. The SILVA ribosomal RNA gene database project: improved data processing and web-based tools. Nucleic Acids Research 41, D590–D596.

Tamura, K., Nei, M., 1993. Estimation of the number of nucleotide substitutions in the control region of mitochondrial DNA in humans and chimpazees. Molecular Biology and Evolution 10, 512–526.

Větrovský, T., Baldrian, P., Morais, D., 2018. SEED 2: a user-friendly platform for amplicon high-throughput sequencing data analyses. Bioinformatics 34, 2292–2294.

## SUPPLEMENTARY TABLES

Table S1. Physical and chemical properties of soils (mean ± SD, n=3) collected from three dairy farms that were used for setting up the microcosms.

| Source | Soil load by livestock [LU] | Texture | pH | N [mg $g^{-1}$ dw] | Ptot [mg $g^{-1}$ dw] | Ctot [mg $g^{-1}$ dw] | Dry matter (%) | C/N |
|---|---|---|---|---|---|---|---|---|
| S | 1.0 | sandy loam | 5.52 (0.04) | 3.03 (0.06) | 0.45 (0.07) | 31.97 (1.57) | 72.5 (0.2) | 12.27 (0.42) |
| B | 0.6 | sandy loam | 4.72 (0.02) | 1.45 (0.09) | 0.73 (0.08) | 15.38 (1.25) | 82.1 (0.5) | 12.37 (0.27) |
| M | 0.4 | sandy loam | 5.16 (0.02) | 2.79 (0.05) | 0.92 (0.02) | 25.65 (0.28) | 83.0 (0.6) | 10.72 (0.28) |

Table S2. TET-resistant bacterial isolates from fresh manure and soil (S, B and M) at the interlayer. The presence of specific TET-r genes, including *tet*(A), *tet*(Y), *tet*(M), *tet*(O), *tet*(Q), *tet*(W), *traN* and *tet*(X) was confirmed for several bacterial isolates by sequencing. *ND* not detected.

| Source | Treatment | Time | Genus assigned | BLAST closest relative and accession number | Identity (%) with BLAST hit | Class | Growth conditions | TET-r gene |
|---|---|---|---|---|---|---|---|---|
| Fresh manure | Fresh manure | T0 | *Acinetobacter* | *Acinetobacter sp. (MH482959)* | 98 | *Gammaproteobacteria* | Chromoagar (TSA+tet), aerobic | ND |
| Fresh manure | Fresh manure | T0 | *Acinetobacter* | *Acinetobacter sp. (DI101487)* | 98 | *Gammaproteobacteria* | Chromoagar (TSA+tet), aerobic | ND |
| Fresh manure | Fresh manure | T0 | *Acinetobacter* | *Acinetobacter sp. (KT907046)* | 99.2 | *Gammaproteobacteria* | TSA (TSA+tet), aerobic | ND |
| Fresh manure | Fresh manure | T0 | *Escherichia* | *E. coli (NR024570)* | 99.5 | *Gammaproteobacteria* | Schaedler (Scha+tet), anaerobic | ND |
| Fresh manure | Fresh manure | T0 | *Escherichia* | *E. coli (HG738867)* | 99.2 | *Gammaproteobacteria* | Schaedler (Scha+tet), anaerobic | ND |
| Fresh manure | Fresh manure | T0 | *Cutibacterium* | *Cutibacterium sp. (MH463780)* | 99.6 | *Actinobacteria* | Schaedler (Scha+tet), anaerobic | ND |
| S | A | T7 | *Bacillus* | *Bacillus sp. (KY805997)* | 99.9 | *Bacili* | Schaedler (Scha+tet), anaerobic | ND |
| S | A | T7 | *Cutibacterium* | *Cutibacterium sp. (MH463780)* | 99.7 | *Actinobacteria* | Schaedler (Scha+tet), anaerobic | ND |
| S | A | T7 | *Dyella* | *D. koreensis (NR043258)* | 99.4 | *Gammaproteobacteria* | Chromoagar (TSA+tet), aerobic | ND |
| S | A | T7 | *Paenibacillus* | *Paenibacillus sp. (NR043258)* | 99.5 | *Bacili* | TSA (TSA+tet), aerobic | ND |
| S | A | T7 | *Paenibacillus* | *P. lautus (NR053258)* | 99.5 | *Bacili* | TSA (TSA+tet), aerobic | ND |
| S | A | T7 | *Paenibacillus* | *Paenibacillus sp. (MH430569)* | 99 | *Bacili* | TSA (TSA+tet), aerobic | ND |
| S | A | T7 | *Paenibacillus* | *Paenibacillus sp. (MF289501)* | 98.9 | *Bacili* | TSA (TSA+tet), aerobic | ND |
| S | A | T7 | *Pseudomonas* | *P. fluorescens (CP010945)* | 99 | *Gammaproteobacteria* | Chromoagar (TSA+tet), aerobic | ND |
| S | A | T7 | *Streptomyces* | *Streptomyces sp. (CM001165)* | 99 | *Actinomycetales* | TSA (TSA+tet), aerobic | ND |
| S | B | T7 | *Bacillus* | *B. toyonensis (JH792135)* | 99.8 | *Bacili* | Schaedler (Scha+tet), anaerobic | ND |
| S | B | T7 | *Variovorax* | *V. paradoxus (AY878410)* | 99.9 | *Betaproteobacteria* | Chromoagar (TSA+tet), aerobic | ND |
| S | C | T7 | *Acinetobacter* | *A. haemolyticus (NZ01000140)* | 99.8 | *Gammaproteobacteria* | Chromoagar (TSA+tet), aerobic | ND |
| S | C | T7 | *Acinetobacter* | *A. gandensis (NR133953)* | 99.6 | *Gammaproteobacteria* | Chromoagar (TSA+tet), aerobic | ND |
| S | C | T7 | *Escherichia* | *E. coli (AE014075)* | 98.2 | *Gammaproteobacteria* | Schaedler (Scha+tet), anaerobic | ND |
| S | C | T7 | *Escherichia* | *E. coli (HG738867)* | 97.8 | *Gammaproteobacteria* | Schaedler (Scha+tet), anaerobic | ND |
| S | C | T7 | *Escherichia* | *E. coli (NR024570)* | 98.5 | *Gammaproteobacteria* | Schaedler (Scha+tet), anaerobic | ND |
| S | C | T7 | *Escherichia* | *E. coli (HG738867)* | 97.9 | *Gammaproteobacteria* | Schaedler (Scha+tet), anaerobic | ND |
| S | C | T7 | *Escherichia* | *E. coli (CP007394)* | 98.9 | *Gammaproteobacteria* | Schaedler (Scha+tet), anaerobic | ND |
| S | C | T7 | *Escherichia* | *E. coli (NR024570)* | 98.9 | *Gammaproteobacteria* | Schaedler (Scha+tet), anaerobic | ND |
| S | C | T7 | *Ochrobactrum* | *Ochrobactrum sp. (MF991910)* | 99.6 | *Alfaproteobacteria* | Chromoagar (TSA+tet), aerobic | ND |
| S | C | T7 | *Pseudomonas* | *Pseudomonas sp. (KY927415)* | 99 | *Gammaproteobacteria* | Chromoagar (TSA+tet), aerobic | ND |
| S | C | T7 | *Rhodococcus* | *Rhodococcus sp. (U27579)* | 99.3 | *Actinomycetales* | TSA (TSA+tet), aerobic | ND |
| S | C | T7 | *Variovorax* | *V. paradoxus (AF532868)* | 99.8 | *Betaproteobacteria* | Chromoagar (TSA+tet), aerobic | ND |
| S | C | T7 | *Variovorax* | *Variovorax sp. (KX665557)* | 98 | *Betaproteobacteria* | Chromoagar (TSA+tet), aerobic | ***tet*(O)** |
| S | D | T7 | *Acinetobacter* | *Acinetobacter sp. (MG255172)* | 98 | *Gammaproteobacteria* | Chromoagar (TSA+tet), aerobic | ***tet*(Y)** |
| S | D | T7 | *Achromobacter* | *A. xylosoxidans (DQ174269)* | 99.5 | *Betaproteobacteria* | Chromoagar (TSA+tet), aerobic | ND |
| S | D | T7 | *Achromobacter* | *Acinetobacter sp. (MH685380)* | 99.6 | *Betaproteobacteria* | Chromoagar (TSA+tet), aerobic | ND |
| S | D | T7 | *Alcaligenes* | *A. faecalis (KF500593)* | 99.9 | *Betaproteobacteria* | Chromoagar (TSA+tet), aerobic | ND |
| S | D | T7 | *Escherichia* | *Escherichia sp. (NZ01000533)* | 99.5 | *Gammaproteobacteria* | Schaedler (Scha+tet), anaerobic | ***tet*(Y)** |
| S | D | T7 | *Shigella* | *Shigella sp. (MI01001411 )* | 99.2 | *Gammaproteobacteria* | Schaedler (Scha+tet), anaerobic | ND |
| S | D | T7 | *Escherichia* | *E. coli (HG738867)* | 98.7 | *Gammaproteobacteria* | Schaedler (Scha+tet), anaerobic | ND |
| S | D | T7 | *Escherichia* | *E. coli (NR024570)* | 99.1 | *Gammaproteobacteria* | Schaedler (Scha+tet), anaerobic | ND |
| S | D | T7 | *Escherichia* | *E. coli (AE014075)* | 99.2 | *Gammaproteobacteria* | Schaedler (Scha+tet), anaerobic | ND |

| | | | | | | | | |
|---|---|---|---|---|---|---|---|---|
| S | D | T7 | *Escherichia* | *E. coli (HG738867)* | 98.5 | *Gammaproteobacteria* | Schaedler (Scha+tet), anaerobic | ND |
| S | D | T7 | *Escherichia* | *E. coli (AE014075)* | 98.4 | *Gammaproteobacteria* | Schaedler (Scha+tet), anaerobic | ND |
| S | D | T7 | *Escherichia* | *E. coli (NR024570)* | 99.8 | *Gammaproteobacteria* | Schaedler (Scha+tet), anaerobic | ND |
| S | D | T7 | *Escherichia* | *E. coli (AE014075)* | 98.3 | *Gammaproteobacteria* | Schaedler (Scha+tet), anaerobic | ND |
| S | D | T7 | *Microbacterium* | *M. paraoxydans (AJ581908)* | 99 | *Actinomycetales* | TSA (TSA+tet), aerobic | ND |
| S | D | T7 | *Pseudomonas* | *Pseudomonas sp. (AP017423)* | 99.8 | *Gammaproteobacteria* | Chromoagar (TSA+tet), aerobic | ND |
| S | D | T7 | *Pseudomonas* | *Pseudomonas sp. (KY927414)* | 96.2 | *Gammaproteobacteria* | TSA (TSA+tet), aerobic | ND |
| S | D | T7 | *Pseudomonas* | *Pseudomonas sp. (KY927415)* | 99.5 | *Gammaproteobacteria* | TSA (TSA+tet), aerobic | ND |
| S | D | T7 | *Pseudomonas* | *Pseudomonas sp. (KY927414)* | 97.7 | *Gammaproteobacteria* | Endoagar (TSA+tet), aerobic | ND |
| S | D | T7 | *Salmonella* | *S. enterica (CP003278)* | 98.5 | *Gammaproteobacteria* | Schaedler (Scha+tet), anaerobic | ND |
| S | D | T7 | *Sphingobacterium* | *Sphingobacterium sp. (MH930040)* | 95.8 | *Sphingobacteria* | TSA (TSA+tet), aerobic | ND |
| S | D | T7 | *Sphingobacterium* | *S. alimentarium (NR108489)* | 100 | *Sphingobacteria* | TSA (TSA+tet), aerobic | ND |
| S | D | T7 | *Sphingobacterium* | *S. alimentarium (MG706020)* | 95.1 | *Sphingobacteria* | TSA (TSA+tet), aerobic | ***tet*(X) and *tet*(O)** |
| S | D | T7 | *Streptococcus* | *S. equinus (NZ01000004)* | 100 | *Bacili* | Schaedler (Scha+tet), anaerobic | ND |
| B | A | T7 | *Bacillus* | *B. mycoides (CM000719)* | 100 | *Bacili* | Schaedler (Scha+tet), anaerobic | ND |
| B | A | T7 | *Bacillus* | *B. mycoides (CM000737)* | 99.5 | *Bacili* | Schaedler (Scha+tet), anaerobic | ND |
| B | A | T7 | *Variovorax* | *V. paradoxus (AF532868)* | 99.8 | *Betaproteobacteria* | Chromoagar (TSA+tet), aerobic | ND |
| B | A | T7 | *Variovorax* | *Variovorax sp. (KX665553)* | 99.3 | *Betaproteobacteria* | TSA (TSA+tet), aerobic | ND |
| B | A | T7 | *Streptomyces* | *Streptomyces sp. (MH796118)* | 99.2 | *Actinomycetales* | TSA (TSA+tet), aerobic | ND |
| B | B | T7 | *Leifsonia* | *Leifsonia sp. (KY033218)* | 99 | *Actinomycetales* | TSA (TSA+tet), aerobic | ND |
| B | C | T7 | *Acinetobacter* | *A. haemolyticus (NZ01000140)* | 99.3 | *Gammaproteobacteria* | Chromoagar (TSA+tet), aerobic | ND |
| B | C | T7 | *Acinetobacter* | *A. gandensis (KM206133)* | 99 | *Gammaproteobacteria* | Endoagar (TSA+tet), aerobic | ***tet*(Y)** |
| B | C | T7 | *Acinetobacter* | *A. junii (EF492020)* | 99.5 | *Gammaproteobacteria* | TSA (TSA+tet), aerobic | ***tet*(Y)** |
| B | C | T7 | *Acinetobacter* | *A. gandensis (KM206132)* | 99.7 | *Gammaproteobacteria* | TSA (TSA+tet), aerobic | ND |
| B | C | T7 | *Acinetobacter* | *A. gandensis (KM206131)* | 98.4 | *Gammaproteobacteria* | Chromoagar (TSA+tet), aerobic | ND |
| B | C | T7 | *Escherichia* | *E. coli (AE014075)* | 98.4 | *Gammaproteobacteria* | Schaedler (Scha+tet), anaerobic | ND |
| B | C | T7 | *Escherichia* | *E. coli (NR024570)* | 98 | *Gammaproteobacteria* | Schaedler (Scha+tet), anaerobic | ND |
| B | C | T7 | *Escherichia* | *E. coli (AE014075)* | 99.1 | *Gammaproteobacteria* | Schaedler (Scha+tet), anaerobic | ND |
| B | C | T7 | *Escherichia* | *E. coli (HG738867)* | 99 | *Gammaproteobacteria* | Schaedler (Scha+tet), anaerobic | ND |
| B | C | T7 | *Escherichia* | *E. coli (AE014075)* | 98.7 | *Gammaproteobacteria* | Schaedler (Scha+tet), anaerobic | ND |
| B | C | T7 | *Escherichia* | *E. coli (NR024570)* | 98.4 | *Gammaproteobacteria* | Schaedler (Scha+tet), anaerobic | ND |
| B | C | T7 | *Escherichia* | *E. coli (HG738867)* | 98.8 | *Gammaproteobacteria* | Schaedler (Scha+tet), anaerobic | ND |
| B | C | T7 | *Escherichia* | *E. coli (NR024570)* | 97.7 | *Gammaproteobacteria* | Schaedler (Scha+tet), anaerobic | ND |
| B | C | T7 | *Escherichia* | *E. coli (AE014075)* | 97.7 | *Gammaproteobacteria* | Schaedler (Scha+tet), anaerobic | ND |
| B | C | T7 | *Microbacterium* | *Microbacterium sp. (KX496339)* | 95.1 | *Actinomycetales* | TSA (TSA+tet), aerobic | ND |
| B | C | T7 | *Pelosinus* | *Pelosinus sp. (KP219716)* | 98.1 | *Negativicutes* | Schaedler (Scha+tet), anaerobic | ***tet*(Y)** |
| B | C | T7 | *Stenothropomonas* | *S. rhizophila (NR121739)* | 99.8 | *Gammaproteobacteria* | Chromoagar (TSA+tet), aerobic | ND |
| B | C | T7 | *Variovorax* | *V. paradoxus (AF532868)* | 99.9 | *Betaproteobacteria* | Chromoagar (TSA+tet), aerobic | ND |
| B | D | T7 | *Achromobacter* | *Achromobacter sp. (MH685380)* | 99.9 | *Betaproteobacteria* | TSA (TSA+tet), aerobic | ND |
| B | D | T7 | *Achromobacter* | *A. xylosoxidans (DQ174269)* | 99.6 | *Betaproteobacteria* | Chromoagar (TSA+tet), aerobic | ND |
| B | D | T7 | *Achromobacter* | *A. xylosoxidans (DQ174269)* | 98.7 | *Betaproteobacteria* | Chromoagar (TSA+tet), aerobic | ND |
| B | D | T7 | *Alcaligenes* | *Alcaligenes. sp. (MF871632)* | 99.6 | *Betaproteobacteria* | Chromoagar (TSA+tet), aerobic | ND |

| | | | | | | | | |
|---|---|---|---|---|---|---|---|---|
| B | D | T7 | *Achromobacter* | *Achromobacter sp. (MH685380)* | 98.9 | *Betaproteobacteria* | TSA (TSA+tet), aerobic | ND |
| B | D | T7 | *Cellulosimicrobium* | *Cellulosimicrobium sp. (MH057217)* | 99.6 | *Actinomycetales* | Schaedler (Scha+tet), anaerobic | ND |
| B | D | T7 | *Enterobacter* | *E. amnigenus (FR717599)* | 99.6 | *Gammaproteobacteria* | Schaedler (Scha+tet), anaerobic | ND |
| B | D | T7 | *Enterobacter* | *E. amnigenus (KC790271)* | 99.5 | *Gammaproteobacteria* | Schaedler (Scha+tet), anaerobic | ND |
| B | D | T7 | *Escherichia* | *E. coli (NR024570)* | 98.6 | *Gammaproteobacteria* | Schaedler (Scha+tet), anaerobic | ND |
| B | D | T7 | *Escherichia* | *E. coli (HG738867)* | 98.8 | *Gammaproteobacteria* | Schaedler (Scha+tet), anaerobic | ND |
| B | D | T7 | *Cronobacter* | *Cronobacter sp. (MG892854)* | 99.2 | *Gammaproteobacteria* | Schaedler (Scha+tet), anaerobic | ND |
| B | D | T7 | *Escherichia* | *E. coli (AE014075)* | 99.3 | *Gammaproteobacteria* | Schaedler (Scha+tet), anaerobic | ND |
| B | D | T7 | *Escherichia* | *E. coli (NR024570)* | 98 | *Gammaproteobacteria* | Schaedler (Scha+tet), anaerobic | ND |
| B | D | T7 | *Escherichia* | *E. coli (AE014075)* | 97.7 | *Gammaproteobacteria* | Schaedler (Scha+tet), anaerobic | ND |
| B | D | T7 | *Escherichia* | *E. coli (HG738867)* | 98 | *Gammaproteobacteria* | Schaedler (Scha+tet), anaerobic | ND |
| B | D | T7 | *Escherichia* | *E. coli (AE014075)* | 99.8 | *Gammaproteobacteria* | Schaedler (Scha+tet), anaerobic | ND |
| B | D | T7 | *Escherichia* | *E. coli (NR024570)* | 98.3 | *Gammaproteobacteria* | Schaedler (Scha+tet), anaerobic | ND |
| B | D | T7 | *Myroides* | *M. odoratimimmus (GU253339)* | 99.1 | *Flavobecteria* | Chromoagar (TSA+tet), aerobic | ND |
| B | D | T7 | *Pseudomonas* | *Pseudomonas sp. (KY927414)* | 100 | *Gammaproteobacteria* | Chromoagar (TSA+tet), aerobic | ND |
| B | D | T7 | *Sphingobacterium* | *S. alimentarium (MG705748)* | 99.9 | *Sphingobacteria* | TSA (TSA+tet), aerobic | ***tet*(X)** |
| B | D | T7 | *Sphingobacterium* | *S. alimentarium (MG705662)* | 93.8 | *Sphingobacteria* | TSA (TSA+tet), aerobic | ND |
| B | D | T7 | *Sphingobacterium* | *S. alimentarium (NR108489)* | 95.6 | *Sphingobacteria* | TSA (TSA+tet), aerobic | ND |
| B | D | T7 | *Sphingobacterium* | *S. alimentarium (MG706020)* | 95.5 | *Sphingobacteria* | TSA (TSA+tet), aerobic | ND |
| B | D | T7 | *Stenotrophomonas* | *Stenotrophomonas sp. (DM065750)* | 99.58 | *Gammaproteobacteria* | Chromoagar (TSA+tet), aerobic | ***tet*(O)** |
| B | D | T7 | *Stenotrophomonas* | *S. maltophilia (DQ230920)* | 100 | *Gammaproteobacteria* | Chromoagar (TSA+tet), aerobic | ND |
| B | D | T7 | *Stenotrophomonas* | *S. maltophilia (AY360340)* | 98.1 | *Gammaproteobacteria* | Chromoagar (TSA+tet), aerobic | ND |
| B | D | T7 | *Stenothropomonas* | *S. rhizophila (NR121739)* | 100 | *Gammaproteobacteria* | Chromoagar (TSA+tet), aerobic | ND |
| M | A | T7 | *Bacillus* | *B. drentensis (KT760403)* | 99.3 | *Bacili* | Schaedler (Scha+tet), anaerobic | ND |
| M | A | T7 | *Clostridium* | *C. subterminale (L37595)* | 99.5 | *Clostridiales* | Schaedler (Scha+tet), anaerobic | ND |
| M | A | T7 | *Clostridium* | *C. subterminale (M59106)* | 99.4 | *Clostridiales* | Schaedler (Scha+tet), anaerobic | ND |
| M | A | T7 | *Rhodococcus* | *Rhodococcus sp. (MG946225)* | 99.6 | *Actinomycetales* | TSA (TSA+tet), aerobic | ND |
| M | A | T7 | *Staphylococcus* | *S. hominis ( NZ01000041)* | 100 | *Bacili* | Schaedler (Scha+tet), anaerobic | ND |
| M | A | T7 | *Variovorax* | *V. paradoxus (AF532868)* | 99.3 | *Betaproteobacteria* | TSA (TSA+tet), aerobic | ND |
| M | C | T7 | *Cronobacter* | *Cronobacter sp. (MG892854)* | 98.8 | *Gammaproteobacteria* | Schaedler (Scha+tet), anaerobic | ND |
| M | C | T7 | *Escherichia* | *E. coli (NR024570)* | 98.4 | *Gammaproteobacteria* | Schaedler (Scha+tet), anaerobic | ND |
| M | C | T7 | *Escherichia* | *E. coli (AE014075)* | 98.6 | *Gammaproteobacteria* | Schaedler (Scha+tet), anaerobic | ND |
| M | C | T7 | *Escherichia* | *E. coli (HG738867)* | 97.6 | *Gammaproteobacteria* | Schaedler (Scha+tet), anaerobic | ND |
| M | C | T7 | *Escherichia* | *E. coli (AE014075)* | 97.7 | *Gammaproteobacteria* | Schaedler (Scha+tet), anaerobic | ND |
| M | C | T7 | *Rhodococcus* | *Rhodococcus sp. (U27579)* | 99.6 | *Actinomycetales* | TSA (TSA+tet), aerobic | ND |
| M | C | T7 | *Rhodococcus* | *Rhodococcus sp. (MG946225)* | 99.7 | *Actinomycetales* | TSA (TSA+tet), aerobic | ND |
| M | C | T7 | *Variovorax* | *V. paradoxus (AF532868)* | 98.8 | *Betaproteobacteria* | Chromoagar (TSA+tet), aerobic | ND |
| M | C | T7 | *Variovorax* | *V. boronicumulans (NR041588)* | 99 | *Betaproteobacteria* | TSA (TSA+tet), aerobic | ND |
| M | C | T7 | *Achromobacter* | *Achromobacter sp. (MH685380)* | 99.4 | *Betaproteobacteria* | Schaedler (Scha+tet), anaerobic | ND |
| M | C | T7 | *Cronobacter* | *Cronobacter sp. (MG892854)* | 98.9 | *Gammaproteobacteria* | Schaedler (Scha+tet), anaerobic | ND |
| M | C | T7 | *Citrobacter* | *Citrobacter sp. (MF477905)* | 98.8 | *Gammaproteobacteria* | Schaedler (Scha+tet), anaerobic | ND |
| M | C | T7 | *Cronobacter* | *Cronobacter sp. (MG892854)* | 98.6 | *Gammaproteobacteria* | Schaedler (Scha+tet), anaerobic | ND |

| | | | | | | | | |
|---|---|---|---|---|---|---|---|---|
| M | C | T7 | *Enterobacter* | *Enterobacter sp. (U39556)* | 99.1 | *Gammaproteobacteria* | Schaedler (Scha+tet), anaerobic | ND |
| M | D | T7 | *Escherichia* | *E. coli (AE014075)* | 99.8 | *Gammaproteobacteria* | Schaedler (Scha+tet), anaerobic | ND |
| M | D | T7 | *Escherichia* | *E. coli (NR024570)* | 98.4 | *Gammaproteobacteria* | Schaedler (Scha+tet), anaerobic | ND |
| M | D | T7 | *Escherichia* | *E. coli (AE014075)* | 97.6 | *Gammaproteobacteria* | Schaedler (Scha+tet), anaerobic | ND |
| M | D | T7 | *Escherichia* | *E. coli (HG738867)* | 98.4 | *Gammaproteobacteria* | Schaedler (Scha+tet), anaerobic | ND |
| M | D | T7 | *Escherichia* | *E. coli (AE014075)* | 99 | *Gammaproteobacteria* | Schaedler (Scha+tet), anaerobic | ND |
| M | D | T7 | *Escherichia* | *E. coli (NR024570)* | 99 | *Gammaproteobacteria* | Schaedler (Scha+tet), anaerobic | ND |
| M | D | T7 | *Labrys* | *Labrys sp. (MH497668)* | 98.6 | *Alfaproteobacteria* | Chromoagar (TSA+tet), aerobic | ND |
| M | D | T7 | *Rhizobium* | *Rhizobium sp. (MH779903)* | 96.8 | *Alfaproteobacteria* | TSA (TSA+tet), aerobic | ***tet*(Y)** |

Table S3. Primers and probes used in this study to amplify tetracycline resistance genes and 16S rRNA (*rrs*) genes.

| Gene | PCR/qPCR | Primers | Sequence 5'-3' | Reference |
|---|---|---|---|---|
| *tet*(A) | PCR | *tet(A) (F)*<br>*tet(A) (R)* | GCTACATCCTGCTTGCCTTC<br>CATAGATCGCCGTGAAGAGG | Ng et al., (2001) |
| *tet*(M) | PCR | *tet(M) (F)*<br>*tet(M) (R)* | GTGGACAAAGGTACAACGAG<br>CGGTAAAGTTCGTCACACAC | Ng et al., (2001) |
| *tet*(O) | PCR | *tet(O) (F)*<br>*tet(O) (R)* | AACTTAGGCATTCTGGCTCAC<br>TCCCACTGTTCCATATCGTCA | Ng et al., (2001) |
| *tet*(Q) | PCR | *tet(Q) (F)*<br>*tet(Q) (R)* | TTATACTTCCTCCGGCATCG<br>ATCGGTTCGAGAATGTCCAC | Ng et al., (2001) |
| *tet*(W) | PCR | *tet(W) (F)*<br>*tet(W) (R)* | GGGAAATTGTTCGGACAGAC<br>AACGGATACCATCCCTGACA | Call et al., (2003) |
| *tet*(X) | PCR | *tet(X)-1*<br>*tet(X)-2* | TTAGCCTTACCAATGGGTGT<br>CAAATCTGCTGTTTCACTCG | Bartha et al., (2011) |
| *tet*(Y) | PCR and qPCR | *tet(Y) (F)*<br>*tet(Y) (R)* | ATTTGTACCGGCAGAGCAAAC<br>GGCGCTGCCGCCATTATGC | Aminov et al., (2002) |
| *traN* (LowGC) | PCR | *V216repF*<br>*V216repR* | AATTGACCGATTTAGTTGTGACC<br>TGCTGATTTGYTTTGGAGATAC | Heuer et al., (2009) |
| *rss* | PCR | *pA*<br>*pH* | AGAGTTTGATCCTGGCTCAG<br>AAGGAGGTGATCCAGCCGCA | Edwards et al., (1989) |
| *tet*(M) | qPCR | *Tet(M) (F)*<br>*Tet(M) (R)*<br>*Probe* | GGTTTCTCTTGGATACTTAAATCAATCR<br>CCAACCATAYAATCCTTGTTCRC<br>FAM-ATGCAGTTATGGARGGGATACGCTATGGY-TAMRA | Peak et al., (2007) |
| *tet*(Q) | qPCR | *Tet(Q) (F)*<br>*Tet(Q) (R)* | AGGTGCTGAACCTTGTTTGATTC<br>GGCCGGACGGAGGATTT | Smith et al., (2004) |
| *tet*(W) | qPCR | *Tet(W) (F)*<br>*Tet(W) (R)* | GCAGAGCGTGGTTCAGTCT<br>GACACCGTCTGCTTGATGATAAT | Smith et al., (2004) |
| *traN* (LowGC) | qPCR | *v216q667f*<br>*v216q741r*<br>*Probe* | GCTTGGCGGTCAGCAATT<br>TTAGGAATAACAATCGCTACACCTTTAC<br>FAM-CTTCTGGCTGCTCCGACACGAAGC-TAMRA | Heuer et al., (2009) |
| *rss* | qPCR | *1108-fw*<br>*1132-rv* | ATGGYTGTCGTCAGCTCGTG<br>GGGTTGCGCTCGTTGC | Amann et al., (1995)<br>Wilmotte et al., (1993) |

## References


Amann, R.I., Ludwig, W., Schleifer, K.H., 1995. Phylogenetic identification and *in situ* detection of individual microbial cells without cultivation. Microbiol. Rev., 59,143–169.

Aminov, R.I., Chee-Sanford, J.C., Garrigues, N., Teferedegne, B., Krapac, I.J., White, B.A., Mackie, R.I., 2002. Development, validation, and application of PCR primers for detection of tetracycline efflux genes of gram-negative bacteria. Appl. Environ. Microbiol., 68, 1786–1793.

Bartha, N.A., Sóki, J., Urbán, E., Nagy, E., 2011. Investigation of the prevalence of *tetQ*, *tetX* and *tetX1* genes in *Bacteroides* strains with elevated tigecycline minimum inhibitory concentrations. Int. J. Antimicrob. Agents, 38, 522–525.

Call, D.R., Bakko, M.K., Krug, M.J., Roberts, M.C., 2003. Identifying antimicrobial resistance genes with DNA microarrays. Antimicrob. Agents. Chemother. 47, 3290–3295.

Edwards, U., Rogall, T., Blöcker, H., Emde, M., Böttger, E.C., 1989. Isolation and direct complete nucleotide determination of entire genes. Characterization of a gene coding for 16S ribosomal RNA. Nucleic Acids Res., 17, 7843–7853.

Heuer, H., Kopmann, C., Binh, C.T.T., Top, E.M., Smalla, K., 2009. Spreading antibiotic resistance through spread manure: characteristics of a novel plasmid type with low %G+C content. Environ. Microbiol., 937–949.

Ng, L.K., Martin, I., Alfa, M., Mulvey, M., 2001. Multiplex PCR for the detection of tetracycline resistant genes. Mol. Cell Probe, 15, 209–215.

Peak, N., Knapp, C. W., Yang, R. K., Hanfelt, M. M., Smith, M. S., Aga, D. S., et al. (2007). Abundance of six tetracycline resistance genes in wastewater lagoons at cattle feedlots with different antibiotic use strategies. Environ. Microbiol., 9, 143–151.

Smith, M.S., Yang, R.K., Knapp, C.W., Niu, Y., Peak, N., Hanfelt, M.M., Galland, J.C., Graham, D.W., 2004. Quantification of tetracycline resistance genes in feedlot lagoons by real-time PCR. Appl. Environ. Microbiol., 70, 7372–7377.

Wilmotte, A., Van der Auwera, F., de Wachter, R., 1993. Structure of the 16S ribosomal RNA of the thermophilic cyanobacterium *Chlorogloeopsis* HTF (*Mastigoclaudus laminosus* HTF) strain PCC7518, and phylogenetic analysis. FEBS Lett., 317, 96–100.

## SUPPLEMENTARY FIGURES

Fig. S1. Boxplot of main soil abiotic properties quantified in the soil interlayer under different treatments at 7 days (T7) and 84 days (T84). Medians, upper and lower quartiles (boxes) and standard deviations (whiskers) were obtained from three soils, each measured in three technical replicates. Treatments are indicated as follows: control soil (A, white), soil + nutrients (B, green), manure + soil (C, red) and manure + γ-irradiated soil (D, yellow). Asterisks indicate significant differences ($p < 0.05$) between individual treatments and the control soil. Differences between C and D treatments were significant for soil pH, nitrogen and phosphorous in both time points.

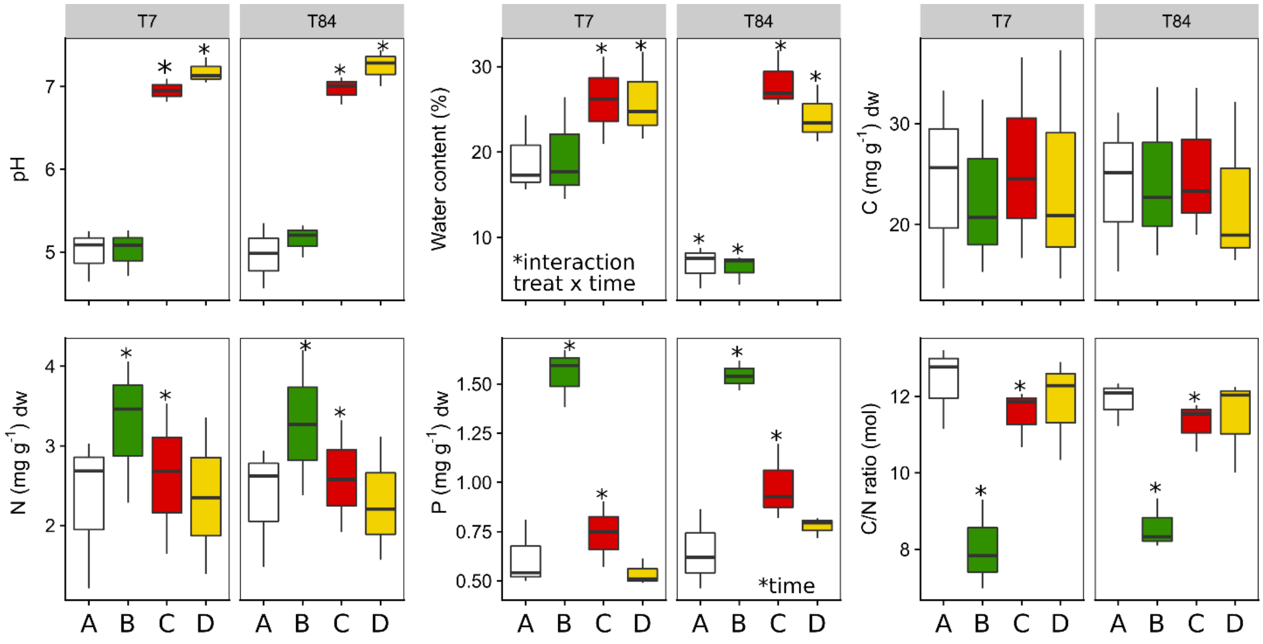

Fig. S2: Venn diagram showing unique (non-overlapping panels) and shared (overlapping panels) TET-resistant OTUs in fresh manure and the S soil at the interlayer at 7 days (T7) and 84 days (T84).

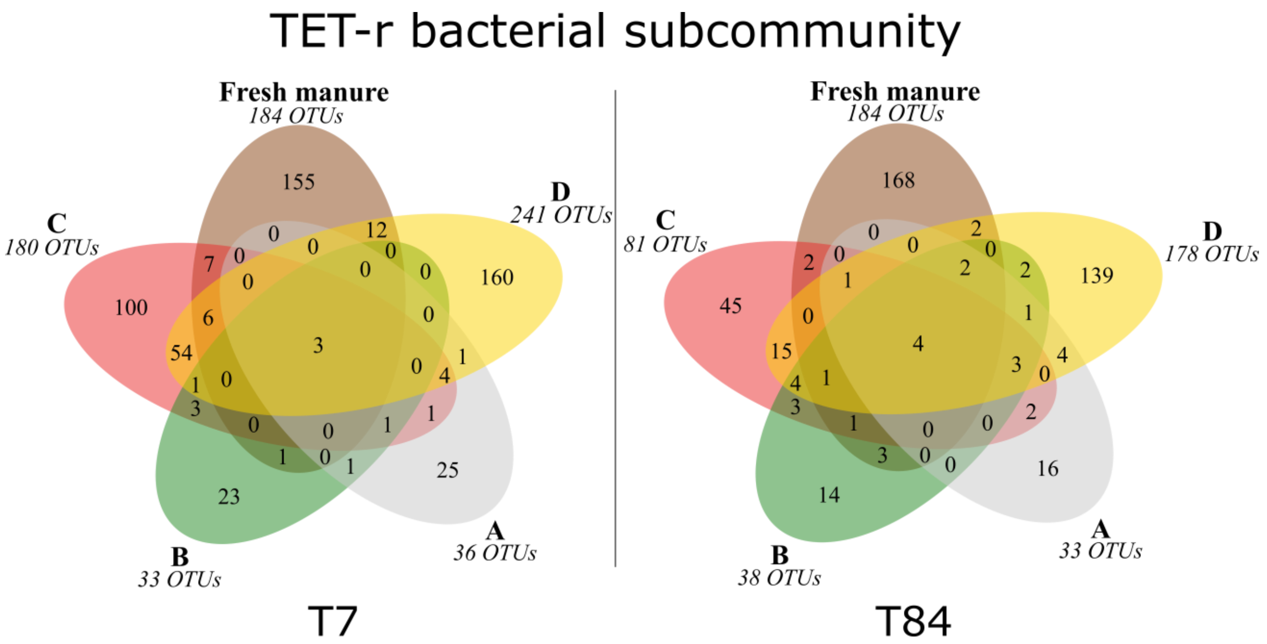

Fig. S3: Relative abundance (in percentage) of the dominant TET-resistant bacterial genera under different treatments (S soil) at 7 days (T7) and 84 days (T84). Average values of three samples per treatment are shown. Standard errors are shown in the table below. DNA was obtained after Nycodenz gradient centrifugation and incubation in presence of TET.

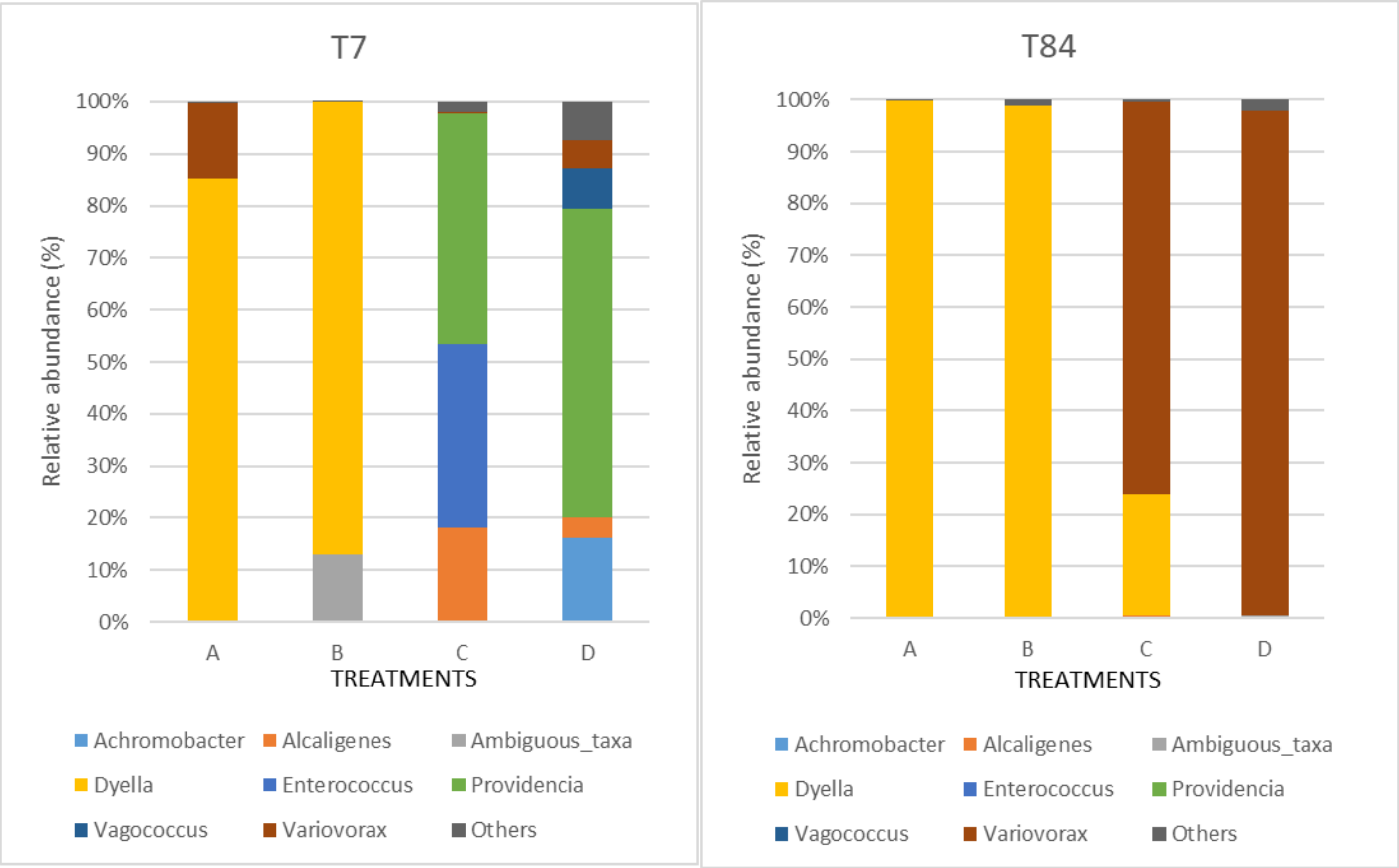


| | SE | | | | | | | |
|---|---|---|---|---|---|---|---|---|
| | T7 | | | | T84 | | | |
| | A | B | C | D | A | B | C | D |
| **Achromobacter** | 0 | 0 | 0 | 2 | 0 | 0 | 0 | 0 |
| **Alcaligenes** | 0 | 0 | 1 | 0 | 0 | 0 | 0 | 0 |
| **Ambiguous_taxa** | 0 | 1 | 0 | 0 | 0 | 0 | 0 | 0 |
| **Dyella** | 2 | 1 | 0 | 0 | 0 | 0 | 0 | 0 |
| **Enterococcus** | 0 | 0 | 1 | 0 | 0 | 0 | 0 | 0 |
| **Providencia** | 0 | 0 | 3 | 3 | 0 | 0 | 0 | 0 |
| **Vagococcus** | 0 | 0 | 0 | 1 | 0 | 0 | 0 | 0 |
| **Variovorax** | 1 | 0 | 0 | 1 | 0 | 0 | 0 | 1 |
| **Others** | 0 | 0 | 0 | 2 | 0 | 0 | 0 | 1 |

Fig. S4: Venn diagram showing unique (non-overlapping panels) and shared (overlapping panels) OTUs in fresh manure and the S soil at the interlayer at 7 days (T7) and 84 days (T84).

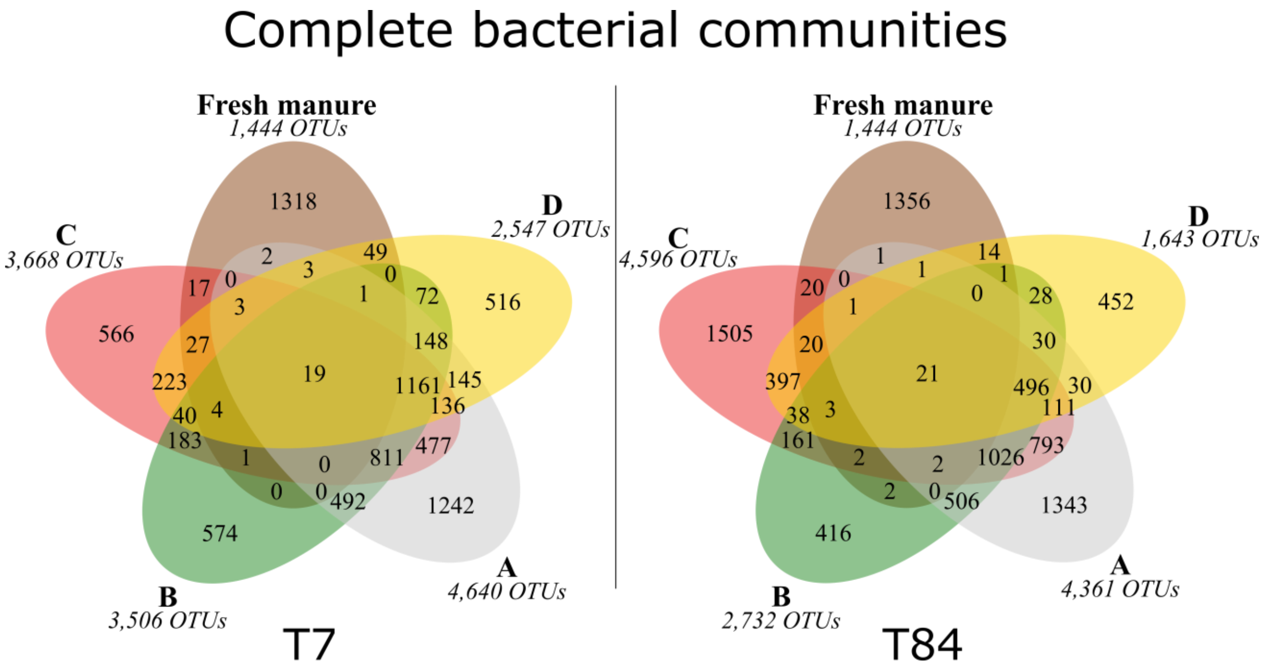

Fig. S5: Relative abundance (in percentage) of the main phyla (class for *Proteobacteria*) under different treatments (S soil) at 7 days (T7) and 84 days (T84). Average values of three samples per treatment are shown. Standard errors are shown in the table below.

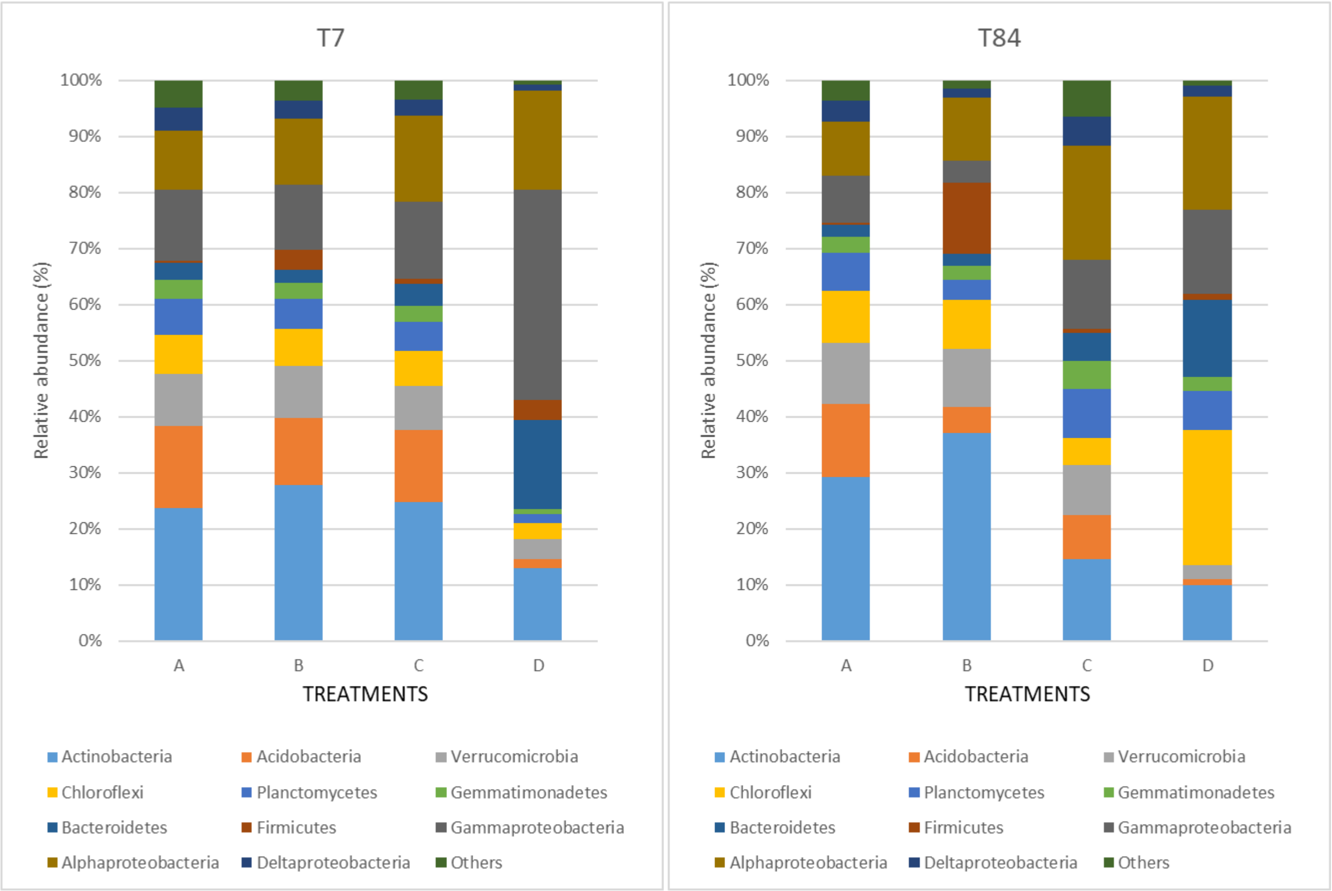


| | SE | | | | | | | |
|---|---|---|---|---|---|---|---|---|
| | T7 | | | | T84 | | | |
| | **A** | **B** | **C** | **D** | **A** | **B** | **C** | **D** |
| **Actinobacteria** | 1 | 1 | 0 | 0 | 3 | 0 | 0 | 0 |
| **Acidobacteria** | 0 | 0 | 0 | 0 | 0 | 0 | 0 | 0 |
| **Verrucomicrobia** | 0 | 0 | 0 | 0 | 1 | 0 | 0 | 0 |
| **Chloroflexi** | 0 | 0 | 0 | 0 | 0 | 0 | 0 | 2 |
| **Planctomycetes** | 0 | 0 | 0 | 0 | 1 | 0 | 0 | 1 |
| **Gemmatimonadetes** | 0 | 0 | 0 | 0 | 0 | 0 | 0 | 0 |
| **Bacteroidetes** | 0 | 0 | 0 | 1 | 0 | 0 | 0 | 1 |
| **Firmicutes** | 0 | 0 | 0 | 0 | 0 | 0 | 0 | 0 |
| **Gammaproteobacteria** | 0 | 0 | 0 | 2 | 0 | 0 | 1 | 0 |
| **Alphaproteobacteria** | 0 | 0 | 1 | 1 | 0 | 0 | 1 | 0 |
| **Deltaproteobacteria** | 0 | 0 | 0 | 0 | 0 | 0 | 0 | 0 |
| **Others** | 0 | 0 | 0 | 0 | 0 | 0 | 0 | 0 |
